# Endoplasmic reticulum stress alters myelin associated protein expression and extracellular vesicle composition in human oligodendrocytes

**DOI:** 10.3389/fmolb.2024.1432945

**Published:** 2024-10-01

**Authors:** Ethan D. Evalt, Saranraj Govindaraj, Madison T. Jones, Nesve Ozsoy, Han Chen, Ashley E. Russell

**Affiliations:** ^1^ Department of Biology, School of Science, The Behrend College, Erie, PA, United States; ^2^ The Transmission Electron Microscopy (TEM) Core, Penn State College of Medicine, Hershey, PA, United States; ^3^ Magee Womens Research Institute, Allied Member, Pittsburgh, PA, United States

**Keywords:** extracellular vesicles, multiple sclerosis, oligodendrocytes, endoplasmic reticulum stress, autophagy

## Abstract

Myelination of the central nervous system is mediated by specialized glial cells called oligodendrocytes (OLs). Multiple sclerosis (MS) is characterized by loss of myelination and subsequent clinical symptoms that can severely impact the quality of life and mobility of those affected by the disease. The major protein components of myelin sheaths are synthesized in the endoplasmic reticulum (ER), and ER stress has been observed in patients with MS. Extracellular vesicles (EVs) have been shown to carry bioactive cargo and have the potential to be utilized as noninvasive biomarkers for various diseases. In the current study, we sought to determine how ER stress in OLs affected the production of key myelination proteins and EV release and composition. To achieve this, tunicamycin was used to induce ER stress in a human oligodendroglioma cell line and changes in myelination protein expression and markers of autophagy were assessed. EVs were also separated from the conditioned cell culture media through size exclusion chromatography and characterized. Significant reductions in the expression of myelination proteins and alterations to autophagosome formation were observed in cells undergoing ER stress. EVs released from these cells were slightly smaller relative to controls, and had strong expression of LC3B. We also observed significant upregulation of miR-29a-3p in ER stress EVs when compared to controls. Taken together, these data suggest that ER stress negatively impacts production of key myelination proteins and induces cells to release EVs that may function to preemptively activate autophagic pathways in neighboring cells.

## 1 Introduction

Oligodendrocytes (OLs) are a specialized type of glial cell found in the central nervous system (CNS) that play a crucial role in facilitating efficient neural communication. OLs are responsible for the formation and maintenance of myelin sheaths, which are comprised of multiple layers of lipid-rich membrane, wrapped around neuronal axons. Myelin sheaths provide electrical insulation for axons and support the fast and effective propagation of electrical impulses with minimal signal loss or degradation. Multiple sclerosis (MS) is a demyelinating autoimmune disorder in which the body’s immune system attacks and degrades the myelin sheath. Without proper insulation, neural electrical signaling is disrupted and leads to a number of neurological deficits, including cognitive, motor, and sensory impairments, inflammation, and neuronal loss (Tafti et al., 2022).

The endoplasmic reticulum (ER) is responsible for synthesizing and folding membrane-bound proteins, as well as proteins to be secreted from the cell (Schwarz and Blower, 2016). Chaperone proteins, including binding immunoglobulin protein (BiP) assist with protein folding and work to prevent protein aggregation (Braakman and Hebert, 2013). If the capacity of the ER to synthesize proteins becomes impaired, the unfolded protein response (UPR), which has been observed in MS, may become activated (Lin et al., 2008; Stone and Lin, 2015). Several ER associated proteins, including Protein Kinase RNA-Like ER kinase (PERK) and inositol-requiring enzyme 1 (IRE1) play major roles in mediating the UPR, which initially aims to promote cellular survival and restore homeostasis (Walter et al., 2018). However, if the cell is unsuccessful at restoring homeostasis, apoptotic pathways will be activated, and the cell will die (Walter et al., 2018).

Several transmembrane proteins, including myelin basic protein (MBP) and proteolipid protein (PLP) (Morell and Quarles, 1999; Lees and Brostoff, 1984), are the major protein components of myelin sheaths and are synthesized in the ER. The membrane-bound protein, 2,3′-cyclic nucleotide 3′-phosphodiesterase (CNPase), plays a major role in myelin formation in OLs (Angelis and Braun, 1994) and is also synthesized in the ER. If OLs experience ER stress, it is likely that production of these key myelination proteins will be downregulated as a result.

ER stress has also been shown to both activate and suppress autophagy in cells (Rashid et al., 2015; Kwon, Kim, and Kim, 2023). Autophagy is an intracellular degradation process that works to clear damaged or unnecessary proteins and organelles from the cell through lysosomal degradation. In the current study we sought to determine how ER stress affected autophagy in OLs.

Further, ER stress has previously been shown to alter the release and composition of extracellular vesicles (EVs) in other cell types, however very little is known about the effects in OLs (Kanemoto et al., 2016; Collett et al., 2018). EVs are small (50–500 nm) lipid-bound particles released from all cells and are present in all biological fluids (Yáñez-Mó et al., 2015). They have been shown to contain proteins, nucleic acids, and lipids that are reflective of the parental cell from which they are derived and can have biologically relevant effects on recipient cells they interact with (Ratajczak et al., 2006; Valadi et al., 2007). EVs released from OLs have previously been shown to mediate changes in their microenvironment by interacting with microglia and neurons and may play important roles in the immune response and pathophysiology of various neurological disorders, including MS (Krämer-Albers, 2020).

In the current study, we sought to begin elucidating the effects of ER stress on OLs, their key myelination proteins, autophagy, and extracellular vesicle profiles.

## 2 Methods

### 2.1 Cell culture

Human oligodendroglioma (HOG) cells were obtained from Millipore Sigma (catalog #SCC163, lot 3427391). The cells were cultured in DMEM (Corning; catalog #10-014-CV) supplemented with 10% fetal bovine serum (VWR; catalog #97068-085) and 1% penicillin-streptomycin (Sigma; Catalog #P4458). For all EV experiments, media supplemented with 10% EV depleted fetal bovine serum (Gibco, A2720801) and 1% penicillin-streptomycin was used. When cells were at least 80% confluent they were trypsinized and centrifuged at 124 × *g* for 5 min. Cells were counted with a Countess™ 3 Automated Cell Counter using trypan blue staining. All experiments used cells from passages 2–10.

### 2.2 Tunicamycin reconstitution and exposure

Tunicamycin (Tocris; 3516) was reconstituted at 10 mg/mL in dimethyl sulfoxide (DMSO) (Chem Cruz; sc-358801). Dilutions were made with cell culture media to obtain concentrations of 1, 5, and 10 μg/mL.

### 2.3 Cell viability assays

To assess cytotoxicity and cell viability after tunicamycin exposure, lactate dehydrogenase (LDH) (Dojindo; CK12) and cell counting kit 8 (CCK8) (Dojindo; CK04) assays were performed, respectively. Cells were seeded at a density of 10,000 cells per well in a 96-well clear bottom black microplate and cultured for 24 h. Media was then gently aspirated from the wells and replaced with 100 μL cell culture media containing 1, 5, or 10 μg/mL tunicamycin or vehicle control (DMSO) for 24 h. At the end of the exposure period, 10 µL LDH Lysis Buffer was added to at least 6 control wells and the plate was returned to the incubator for 30 min to induce maximum cell death. At the conclusion of the incubation period, 50 µL media from each well was transferred to a new 96 well plate, and 50 µL LDH Assay Buffer was added to each well containing the conditioned cell culture media. The plate was incubated for 30 min at room temperature in the dark, 50 µL LDH Stop Solution was added to each well, and then the absorbance was read at 490 nm with a Synergy H1 (Biotek) microplate reader. Meanwhile, to the cell culture plate, 10 µL CCK8 solution was added to each well containing cells and the plate was returned to the cell culture incubator for 1 h. The plate was then read at 450 nm using the same plate reader.

### 2.4 Collection of conditioned cell culture media and cell pellets

Cells were seeded at a density of 2.3 × 10^6^ in T175 flasks with 25 mL of media for 48 h, after which the media was gently aspirated and discarded, and the cells were washed with PBS. Next, 25 mL of EV-depleted media containing 10 μg/mL tunicamycin or vehicle control was added to the flask for 24 h.

After 24 h, the conditioned cell culture media was collected and centrifuged at 124 × g for 5 min at 4°C. The supernatant was transferred to a new tube and centrifuged at 2,000 × g for 20 min at 4°C. The supernatant was then transferred to an ultrafiltration unit (Sigma-Aldrich; UFC900308) and centrifuged at 4,000 × *g* for 90 min at 4°C. The flow through was discarded and the retentate was transferred to a 1.5 mL microfuge tube and the total volume was adjusted to 500 µL with 0.22 µm filtered PBS, and stored at −80°C.

Simultaneously, after the conditioned cell culture media was removed from the flask, cells were rinsed with PBS and trypsinized. After pelleting, cells were transferred to a 1.5 mL microfuge tube and washed with PBS. The cell pellet was stored at −80°C.

### 2.5 Size exclusion chromatography (SEC)

EVs were separated from the conditioned cell culture media as previously described (Jones et al., 2022). Briefly, retentate samples were thawed on ice while SEC columns (IZON; SP1) were warmed to room temperature. Using the IZON Automatic Fraction Collector (AFC), SEC columns were flushed with 15 mL 0.22 µm filtered PBS, then the 500 µL retentate sample was placed on the column. The AFC automatically dispelled the void volume into a waste basin, then 8 fractions of 500 µL each were collected. Fractions 1–4 (the EV fractions) and fractions 5–8 (the protein fractions) were pooled, respectively, and transferred to ultrafiltration units (Sigma-Aldrich; UFC200324) and centrifuged for 1 h and 45 min at 3,500 × *g* at 4°C, or until the retentate volume was 100 µL. The flow through was discarded and the retentate transferred to a new 1.5 mL microfuge tube and stored at −80°C.

### 2.6 Western blot

All cell pellets used for western blotting were lysed with 2 mL RIPA buffer (1% Tris (1M) pH 7.4, 0.01% sodium dodecyl sulfate, 1% deoxycholate, 1% NP-40, 0.89% NaCl in deionized water) containing 1x protease/phosphatase inhibitor (Cell Signaling Technology; 5872S). Protein concentration was determined via BCA assay (Thermo Scientific; 23225) as per the manufacturer’s instructions. Samples containing 20 µg protein (or 15 µL total volume for all EV and protein fractions), RIPA buffer, and either 4× Laemmli buffer (Bio-Rad; 1610747) or dithiothreitol (DTT) reducing agent (IBI Scientific; IB21040) were boiled at 95°C for 5 min, briefly centrifuged at 10,000 × *g* for 20 s, and placed on ice. Samples and protein standard ladder (Bio-Rad; 1610374) were then loaded onto a 10% Tgx Stain-Free™ FastCast™ polyacrylamide gel (Bio-Rad; 1610183) and run at 200V for 30–50 min with 1X running buffer (10× running buffer; 3.3% Tris base, 14.4% glycine, 1% sodium dodecyl sulfate in deionized water). Protein was transferred to a PVDF membrane (Thermo Fisher Scientific; 88518) at 100 V for 30 min at 4°C in 1X transfer buffer (0.303% Tris base, 1.44% glycine, 20% methanol in deionized water).

Blots were blocked with 5% powdered milk in 1× TBS-Tween (0.24% Tris base, 0.8% NaCl, 0.1% Tween-20 in deionized water) for 1 h at room temperature. Blots were then incubated overnight at 4°C with their respective primary antibody diluted in blocking buffer. Antibodies for the following proteins and their dilutions were utilized, with those run under reducing conditions (DTT) marked with an asterisk: IRE1α (Cell Signaling Technology, 3294, 1:1000), BiP (Cell Signaling Technology, 3177, 1:1000), RL90/PDI (Cell Signaling Technology, 3501, 1:1000), ERO1-α (Cell Signaling Technology, 3264, 1:1000), Calnexin (Abcam, ab22595, 1:1000), CD63 (BD Biosciences, 556019 1:1000), CD9 (BioLegend, 312102, 1:500), CD81 (Santa Cruz, sc-23962, 1:500), Beclin-1 (Abcam, ab114071, 1:1000), ^*^ATG5 (Novus, NB110-53818SS, 1:500), LC3B (Novus, NB100-2220SS, 1:500), ^*^MBP (Novus, NBP1-05203, 1:2000), ^*^PLP (Cell Signaling Technology, 28702S, 1:1000), CNPase (Abcam, ab6319, 1:1000), GAPDH (Thermo Fisher Scientific, MA5-15738, 1:5000), and β-Actin (Santa Cruz, sc-69879, 1:500).

The following day, blots were washed 3× in TBS-Tween for 5 min each, and then incubated in the appropriate HRP-linked secondary antibody, anti-rabbit (Cell Signaling Technology, 7074V) or anti-mouse (Cell Signaling Technology, 7076V), diluted in blocking buffer for 1 h at room temperature. Blots were then washed 3× in TBS-Tween for 5 min each and then incubated in 5 mL Clarity Western ECL Substrate (Bio-Rad; 1705061) for 5 min. Blots were imaged using the Bio-Rad ChemiDoc MP Imaging System and analyzed with Image Lab Software Version 6.1.0 build 7 (Bio-Rad).

### 2.7 Nanoparticle tracking analysis

EV and protein fraction samples were obtained from −80°C and thawed on ice. The ZetaView nanoparticle tracking analysis (NTA) instrument and software were turned on to initialize the experiment. The instrument was rinsed with particle-free water to rinse the cell. After checking the cell quality, the cell was filled with the 100 nm polystyrene alignment bead suspension (1:500,000 dilution) and auto alignment and focus optimizations were completed. Polystyrene beads were rinsed from the cell by injecting particle-free water into the injection port to wash the cell. Samples were diluted (1:1000) with 0.22 µm filtered water and the dilution factor was entered into the program. 1 mL of sample was inserted into the cell with setting sensitivity to 80 and shutter 150. The temperature was set to 23°C and a 488 nm laser was used to run the data collection. This procedure was repeated to test all the samples.

### 2.8 Transmission electron microscopy

10 µL of each sample was pipetted onto a 400-mesh copper grid with carbon-coated formvar film and incubated for 1 min. Formvar-carbon coated grids were glow discharged just before use to increase their hydrophilicity. Excess liquid was removed by paper blotting. The grid was briefly placed on 10 µL of 1% uranyl acetate for 1 min, followed by paper blotting to remove excess liquid. The grid was allowed to dry and was examined the same day, viewed in a JEOL JEM1400 Transmission Electron Microscope (JEOL USA Inc., Peabody, MA, United States) located at the Penn State College of Medicine (RRID Number: SCR_021200).

### 2.9 RNA isolation

RNA was isolated from cell lysates using the RNeasy Mini Kit (Qiagen; 74104) according to the manufacturer’s instructions. Briefly, 350 µL RLT Buffer was added to the cell pellet, followed by 350 µL 70% ethanol and pipette mixed. The lysate was then transferred to an RNeasy Mini spin column and centrifuged at 12,000 × *g* for 15 s. The flow-through was discarded and 700 µL Buffer RW1 was added to the spin column and centrifuged for 15 s at 12,000 × *g*. The flow-through was discarded and 500 µL Buffer RPE was added to the spin column and centrifuged for 15 s at 12,000 × *g*. The flow-through was discarded and 500 µL Buffer RPE was added to the spin column and centrifuged for 2 min at 12,000 × *g*. The RNeasy spin column was transferred to a new 1.5 mL collection tube and 30 µL RNase-free water was added directly to the spin column membrane and centrifuged for 1 min at 12,000 × *g* to elute the RNA.

RNA was isolated from EV and protein fractions using the miRNeasy Serum/Plasma Advanced Kit (Qiagen; 217204) according to the manufacturer’s instructions. Briefly, 60 µL Buffer RPL containing synthetic spike-in mix (Qiagen; 339390) was added to each sample and vortexed, followed by a 3-minute incubation period at room temperature. Next, 20 µL Buffer RPP was added and the samples were again vortexed and incubated for 3 min at room temperature. The samples were then centrifuged at 12,000 × g for 2 min, after which the supernatant was transferred to a new tube and 1 volume of isopropanol was added and the sample was vortexed. The entire volume was transferred to a RNeasy UCP MinElute column and centrifuged at 12,000 × g for 15 s. The column was then sequentially washed with Buffer RWT, Buffer RPE, and 80% ethanol and centrifuged at 12,000 × g for each washing step. 20 μL RNase-free water was used to elute the RNA from the column. RNA concentrations and purity for all samples were measured using a NanoDrop™ One (Thermo Fisher Scientific) spectrophotometer.

### 2.10 cDNA synthesis and PCR

cDNA was synthesized using the QuantiTect Reverse Transcription Kit (Qiagen; 205311) as per the manufacturer’s instructions. First, genomic DNA was eliminated by combining the gDNA Wipeout Buffer with the template RNA and RNase-free water and incubating the sample in a C1000 Touch Thermal Cycler (Bio-Rad) at 42°C for 2 min. Next, master mix containing the Quantiscript Reverse Transcritpase, 5× Quantiscript RT Buffer, and RT Primer Mix was added to each sample. Samples were then placed in the thermocycler for 15 min at 42°C, 3 min at 95°C, then cooled to 4°C.

cDNA for miRCURY LNA miRNA PCR Assay was made using the miRCURY RT kit (Qiagen; 339340) as per the manufacturer’s instructions. Briefly, RNA was thawed on ice and combined with a master mix containing 5× miRCURY RT Reaction Buffer, RNase-free water, 10× miRCURY RT Enzyme Mix, and synthetic RNA spike-ins. Samples were placed in a thermocycler for 60 min at 42°C, 5 min at 95°C, then immediately cooled to 4°C.

qPCR was then performed using the QuantiNova SYBR Green PCR Kit (Qiagen; 208052). cDNA was diluted 1:10 and combined with a master mix containing QuantiNova SYBR Green Master Mix, RNase-free water, and the appropriate primer (Qiagen; 249990), HS-ACTB (GeneGlobe ID; SBM0837585), HS-MOBP (GeneGlobe ID: SBH0016076), HS-CNPase (GeneGlobe ID; SBH0193102), HS-GALC (GeneGlobe ID; SBH0206880), and HS-ERBB4 (GeneGlobe ID; SBH0602732), on a Hard-Shell 384-well PCR plate (Bio-Rad; HSP3805). PCR was performed using a CFX Opus 384 Real Time PCR System (Bio-Rad) with the following steps; 2 min initial heat activation at 95°C, followed by 40 cycles of 5 s denaturation at 95°C and 10 s annealing/extension at 60°C, ending with samples held at 4°C.

For assessment of miRNAs, the miCURY LNA™ SYBR green PCR Kit (Qiagen; 339346) was used. cDNA was diluted 1:10 for cell lysate samples and 1:5 for EV and protein fraction samples and was combined with a master mix containing 2x miRCURY SYBR Green Master Mix, RNase-free water, and the appropriate primer (Qiagen; 339306), hsa-miR-29a-3p primer (GeneGlobe ID; YP00204698), cel-miR-39-3p (GeneGlobe ID; YP00203952), and U6 snRNA (GeneGlobe ID; YP02119464) on a Hard Shell 384-well PCR plate (Bio-Rad; HSP3805). PCR was performed using a CFX Opus 384 Real Time PCR System (Bio-Rad) with the following steps; 2 min initial heat activation at 95°C, followed by 40 cycles of 10 s denaturation at 95°C and 60 s annealing/extension at 56°C, ending with samples held at 4°C.

### 2.11 Autophagy staining

To detect activation of autophagy in live cells DAPGreen (Dojindo; D676-10) was used. 10,000 cells per well were seeded in a 96-well black walled microplate and cultured for 24 h. Media was gently aspirated and replaced with 100 μL cell culture media containing 0.5 μmol/L DAPGreen. Thirty minutes later the supernatant was removed, and the cells were washed with cell culture media twice, followed by the addition of 100 μL cell culture media containing 10 μg/mL tunicamycin or vehicle control. The plate was returned to the cell culture incubator for 24 h and then read using a Synergy H1 (Biotek) microplate reader with an excitation of 450 nm and emission of 535 nm.

### 2.12 Statistical analysis

All experiments were repeated at least three times with *n* = 3–6 flasks or wells per treatment and results are reported as mean ± SEM. Data were analyzed using GraphPad Prism 10.10 Software (Dotmatics) using an unpaired t-test or a one-way ANOVA with Dunnett’s *post hoc* test. Significance was established with a *p* value < 0.05; significance presented as ^*^ *p* < 0.05, ^**^ *p* < 0.01, ^***^ *p* < 0.001, ^****^ *p* < 0.0001.

## 3 Results

### 3.1 Cell viability is impacted by tunicamycin treatment

**FIGURE 1:**
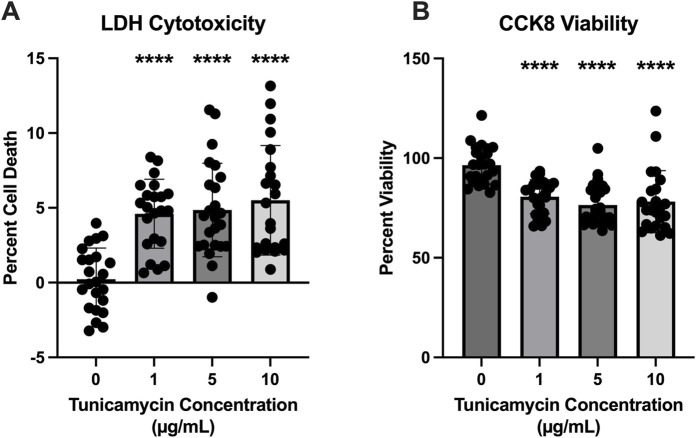
Cell viability is reduced 24 h after tunicamycin exposure. Assessment of cell death and viability after a 24-hour exposure period to various concentrations of tunicamycin. **(A)** Lactate dehydrogenase assay for cytotoxicity [F_(3, 87)_ = 16.20, *p* < 0.0001]; **(B)** CCK8 for cell viability [F_(3, 90)_ = 15.86, *p* < 0.0001]. Although statistically significant cell death and reduced viability was observed, it was not substantial, so 10 μg/mL tunicamycin was chosen for downstream experiments. ^****^ *p* < 0.0001.

To determine the optimal concentration of tunicamycin to induce ER stress without a substantial amount of cell death, LDH cytotoxicity and CCK8 viability assays were performed. While statistically significant cell death was induced (Figure 1A) at all three concentrations of tunicamycin exposure (1, 5, and 10 μg/mL), it was not a substantial amount of cell death (4.601%, 4.858%, and 5.505%, respectively). Similarly, cell viability was statistically significant with lower viability in the tunicamycin exposed cells compared to the vehicle control (Figure 1B), average viability was still quite high with 80.64% viability in the 1 μg/mL tunicamycin treated group, 76.41% in the 5 μg/mL group, and 78.14% in the 10 μg/mL group.

### 3.2 Evidence of ER stress induction

**FIGURE 2:**
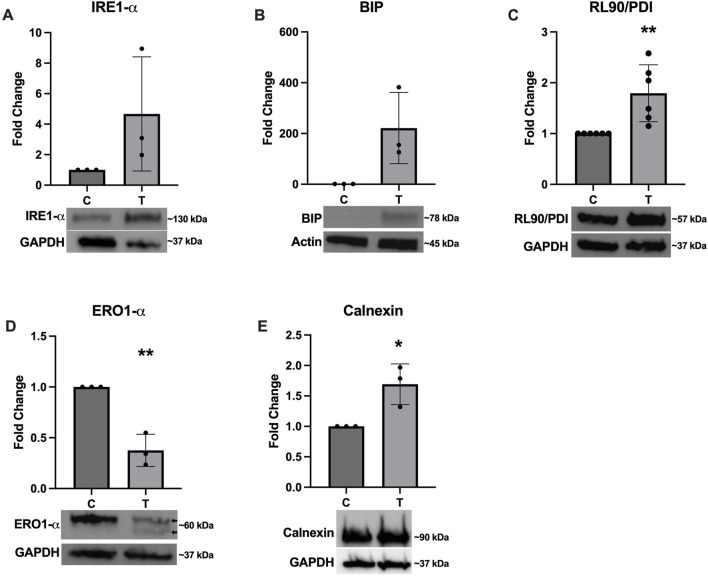
Expression of ER stress associated proteins after tunicamycin exposure. Western blotting was performed after 24-hour exposure to 0 or 10 μg/mL tunicamycin. Expression of **(A)** IRE1-α was not significantly changed. **(B)** BIP was upregulated and almost reached statistical significance [*t* (4) = 2.723, *p* = 0.0528]. **(C)** RL90/PDI [*t* (10) = 3.468, *p* = 0.006] and **(E)** calnexin [*t* (4) = 3.587, *p* = 0.0230] were significantly upregulated, while **(D)** ERO1-α [*t* (4) = 6.801, *p* = 0.0024] was significantly downregulated and displayed double banding in the tunicamycin exposed samples (arrows). ^*^ *p* < 0.05; ^**^ *p* < 0.01.

To confirm successful induction of ER stress, several western blots were performed to assess expression of various proteins with and without 10 μg/mL tunicamycin exposure. Figure 2 depicts a non-significant trend of increased expression of IRE1-α (Figure 2A) and BIP (Figure 2B), with BIP nearing significance (*p* = 0.0528). RL90/PDI (Figure 2C) and Calnexin (Figure 2E) were significantly upregulated while ERO1-α (Figure 2D) was significantly downregulated. Double banding was also observed in ERO1-α, likely indicating redox changes in the ER in response to ER stress (Sevier et al., 2007).

### 3.3 EV characterization

**FIGURE 3:**
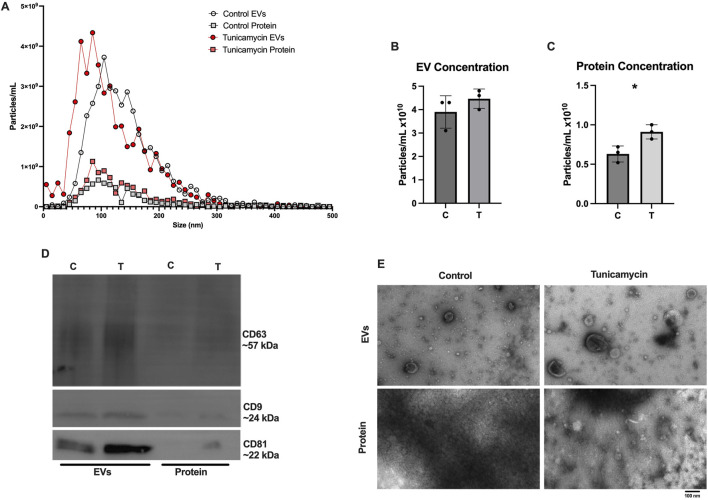
Characterization of Extracellular vesicle profiles. After 24-hour exposure to 0 or 10 μg/mL tunicamycin, EVs were separated and profiled. **(A)** NTA shows slightly smaller particles released from the tunicamycin exposed cells relative to control exposed. **(B)** No difference in overall particle concentration in EVs was observed, but **(C)** significantly more particles were counted in the tunicamycin exposed protein fraction, relative to control [*t* (4) = 3.575, *p* = 0.0233]. **(D)** EVs express the expected markers, CD63, CD9, and CD81 as well as **(E)** the expected morphology in the EV fractions, with no EVs observed in the protein fractions. ^*^ *p* < 0.05.

As recommended by the International Society for Extracellular Vesicles, EVs were characterized based on their size and quantity, protein composition, and morphology (Welsh et al., 2024). In line with previous results published from our lab (Jones et al., 2022), SEC effectively separates EVs from extracellular proteins released in the cell culture media. Figure 3A depicts NTA results for control EVs, control protein, tunicamycin EVs, and tunicamycin protein. Tunicamycin-exposed cells appear to release slightly smaller EVs relative to the control EVs, whereas the particles in the protein fraction do not appear to differ in size. The overall concentration of particles counted in the control and tunicamycin-EV fractions were not significantly different from one another (Figure 3B). However, there were significantly more particles counted in the tunicamycin-protein fraction relative to control (Figure 3C). Western blotting indicated the canonical EV markers, CD63, CD9, and CD81 were present in the EV fractions, with very limited expression in the protein fraction (Figure 3D). It also appears that there may be higher expression of these proteins in the tunicamycin-EVs relative to control. Electron microscopy further supports successful separation of EVs and extracellular proteins, with EVs of the expected morphology and size observed in the EV fractions, and no evidence of their presence in the protein fraction (Figure 3E).

**FIGURE 4:**
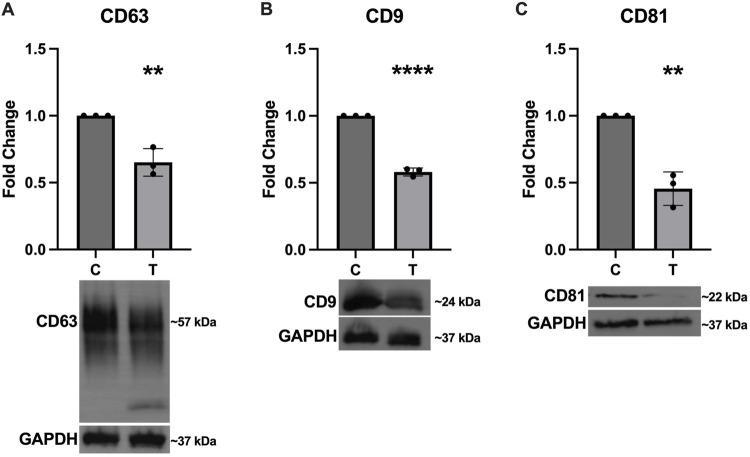
Intracellular expression of EV markers decreases in response to ER stress. After 24-hour exposure to 0 or 10 μg/mL tunicamycin, intracellular expression of **(A)** CD63 [*t* (4) = 5.858, *p* = 0.0042], **(B)** CD9 [*t* (4) = 23.97, *p* < 0.0001], and **(C)** CD81 [*t* (4) = 7.553, *p* = 0.0016] were all significantly downregulated in the tunicamycin exposed cells. ^**^ *p* < 0.01; ^****^ *p* < 0.0001.

In addition to assessing expression of the canonical EV markers in the SEC fractions, we also probed the cell lysates to determine how ER stress affects cellular expression of these proteins. Figure 4 shows significantly decreased expression of CD63 (Figure 4A), CD9 (Figure 4B), and CD81 (Figure 4C).

### 3.4 Autophagy activation is impacted by ER stress

**FIGURE 5:**
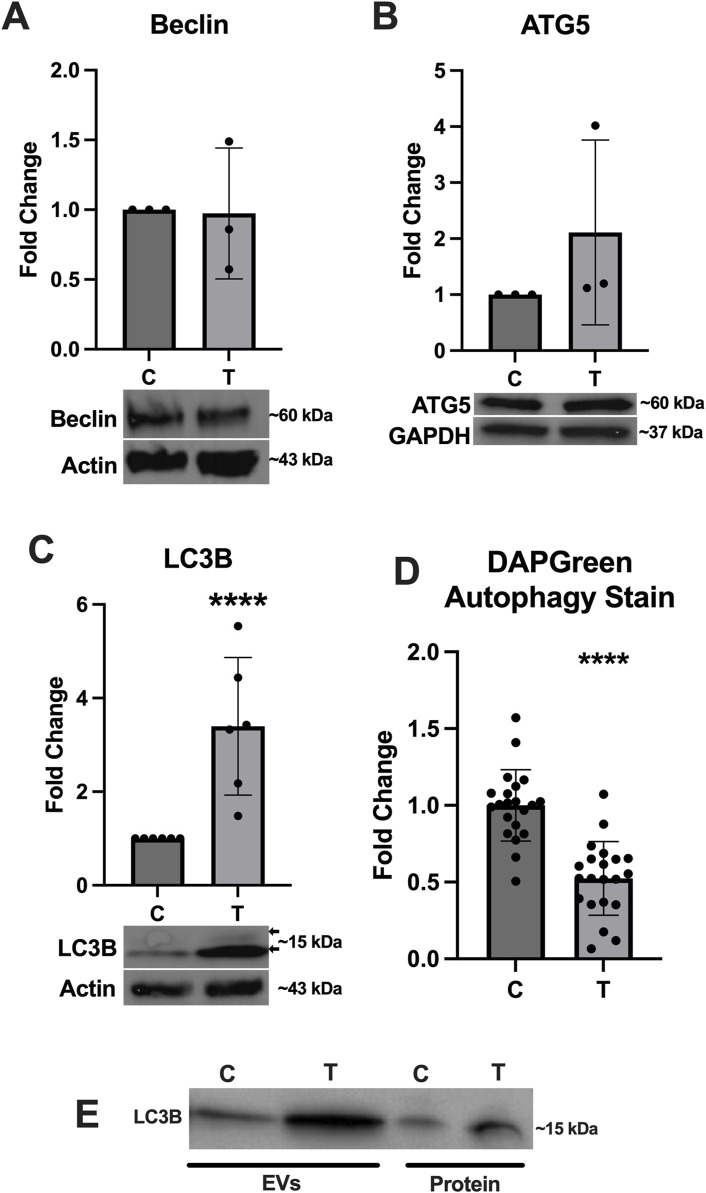
Autophagy associated protein expression changes in response to ER stress. **(A)** Beclin and **(B)** ATG5 were not significantly different between cells exposed to 0 and 10 μg/mL tunicamycin for 24-hour. **(C)** LC3B was significantly upregulated in the tunicamycin exposed cell lysates [*t* (10) = 3.991, *p* = 0.0026]. **(D)** Autophagosome formation was significantly downregulated in the tunicamycin exposed cells [*t* (40) = 6.508, *p* < 0.0001]. **(E)** LC3B was also observed in the EV and protein fractions from both 0 and 10 μg/mL exposed cells. ^****^ *p* < 0.0001.

Because the activation of autophagy is closely linked to ER stress, we assessed expression of several key proteins associated with this pathway. Expression of Beclin (Figure 5A) and ATG5 (Figure 5B) was not significantly altered, however expression of LC3B (Figure 5C) was significantly upregulated and a doublet was observed indicating the conversion of LC3I to LC3II (arrows in Figure 5C). The DAPGreen autophagy assay indicated a significant downregulation in autophagy activation in ER stressed cells compared to controls (Figure 5D). We also probed the EV and protein fractions to determine if any autophagy proteins were present and of those we assessed, only LC3B was observed and appeared to be stronger in the EV and protein fractions from tunicamycin exposed cells (Figure 5E).

### 3.5 Proteins associated with myelination are downregulated after ER stress

**FIGURE 6:**
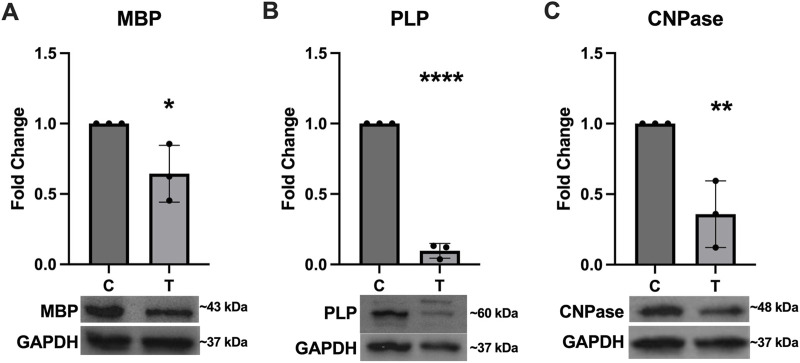
Myelination protein expression is downregulated in response to ER stress. The myelination proteins **(A)** MBP [*t* (4) = 3.056, *p* = 0.0378], **(B)** PLP [*t* (4) = 29.84, *p* < 0.0001], and **(C)** CNPase [*t* (4) = 4.797, *p* = 0.0093] were all significantly downregulated after 24-hour exposure to 10 μg/mL tunicamycin compared to control cells. ^*^ *p* < 0.05; ^**^ *p* < 0.01; ^****^ *p* < 0.0001.

**FIGURE 7:**
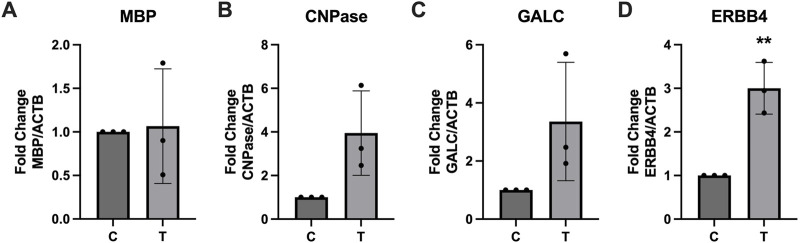
Myelination associated gene expression is altered in response to ER stress. After 24-hour exposure to 0 or 10 μg/mL tunicamycin, **(A)** MBP, **(B)** CNPase, and **(C)** GALC showed no significant changes in gene expression, while **(D)** ERBB4 showed significant increase [*t* (4) = 5.835, *p* = 0.0043]. ^**^ *p* < 0.01.

Expression of several key myelination proteins was also assessed after ER stress (Figure 6). MBP (Figure 6A), PLP (Figure 6B) and CNPase (Figure 6C) were all found to be significantly downregulated after ER stress. We also probed the EV and protein fractions for expression of these proteins but did not detect their presence in any fraction (data not shown). Expression of genes associated with myelination were also assessed using RT-qPCR (Figure 7). No statistically significant changes in expression of MBP (Figure 7A) or GALC (Figure 7C) were observed. A slight increase in CNPase (Figure 7B), that trended towards significance (*p* = 0.0577) was observed. Expression of ERBB was significantly upregulated in tunicamycin-exposed cells relative to control (Figure 7D).

### 3.6 miR-29a-3p expression is elevated in EVs released from tunicamycin exposed cells

**FIGURE 8:**
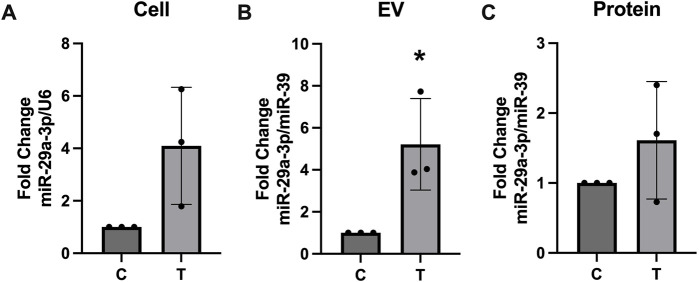
miR-29a-3p expression is upregulated in EVs after tunicamycin exposure. **(A)** Cell lysates and **(C)** protein fractions did not show significant differences in miR-29a-3p expression after 24-hour exposure to 10 μg/mL tunicamycin when compared to controls. **(B)** miR-29a-3p was significantly upregulated in the EV fraction after 10 μg/mL tunicamycin exposure [*t* (4) = 3.350, *p* = 0.0286]. ^*^ *p* < 0.05.

Using Targetscan.org we identified miR-29a-3p as a potential miRNA that could interact with several myelination (PLP, MOG, MPZL3) and autophagy (ATG9, ATG14) related proteins. Expression of miR-29a-3p was assessed in the cellular lysates, EV, and protein fractions of control and tunicamycin exposed cells. While its expression was slightly increased in the cell lysates (Figure 8A) and protein fraction (Figure 8C), it was not statistically significant. Expression of miR-29a-3p in the EVs released from tunicamycin exposed cells was significantly upregulated relative to controls (Figure 8B).

## 4 Discussion

In the current study we first aimed to determine the effects of tunicamycin exposure on the expression of ER stress associated proteins, including IRE1-α, BIP, RL90/PDI, ERO1-α, and calnexin in OLs. (Saito et al., 2011). Under normal conditions, BIP is typically bound to IRE1-α, but during ER stress BIP dissociates and binds to unfolded and misfolded proteins and IRE1-α is phosphorylated and activated (Kimata et al., 2003; Junjappa et al., 2018). Interestingly, and although not statistically significant, we see upregulation in the overall expression of both IRE1-α and BIP after 24 h of tunicamycin-induced ER stress in OLs (Figures 2A, B). PDI is known to oxidize disulfide bonds for protein substrates located in the ER (Munro and Pelham, 1987; Capitani and Sallese, 2009; Schwaller et al., 2003). In an effort to decrease the amount of misfolded proteins accumulating during ER stress, PDI oxidizes misfolded proteins, allowing them to revert back to their native confirmation (Jha et al., 2021). This process is balanced by ERO1-α which reoxidizes PDI (Jha et al., 2021). We observed significant upregulation of PDI and significant downregulation of ERO1-α (Figures 2C, D, respectively). Because tunicamycin exposure results in misfolded proteins accumulating in the ER, it is plausible that PDI is working to mitigate the misfolded proteins through their oxidation; typically, PDI would become reoxidized by ERO1-α, however if its expression is decreased and PDI is left in a reduced state, the cell may be compensating by increasing production of PDI. Further, changes in the oxidation state of ERO1-α have previously been described and observed as double banding on immunoblots (Sevier et al., 2007). These observations further support successful induction of ER stress after tunicamycin treatment in these cells. We also observed a significant increase in expression of calnexin after 24 h of tunicamycin exposure, which has previously been shown to be associated with increased rates of cellular apoptosis (Guérin et al., 2008). We did observe statically significant increases in cell death after tunicamycin exposure (Figure 1A) which could be due, in part, to the activation of calnexin mediated apoptotic pathways. Taken together, these data suggest that ER stress was indeed activated in OLs after 24-hour exposure to 10 μg/mL tunicamycin.

Literature on the impact of ER stress on EV release is conflicting with some studies showing decreased particle counts, while others show increased particle counts (Fukuoka et al., 2023; Collett et al., 2018). Our data suggest that in OLs, ER stress does not significantly impact particle quantities in EV fractions separated through SEC (Figure 3B) but does significantly increase the number of particles quantified in protein fractions. It is possible that these cells are releasing more proteins and protein aggregates into the extracellular space that are not encapsulated in vesicles as a result of ER stress. We also observed that particles in the EV fractions were slightly smaller when released from cells undergoing ER stress compared to EVs from control cells, which may suggest activation of different EV biogenesis pathways (Figure 3A). Both control and ER stressed cells release EVs expressing the canonical EV markers CD63, CD9, and CD81, with EVs derived from ER stressed cells appearing to have higher expression of these markers relative to EVs from control cells (Figure 3D), however ER stressed cells downregulate intracellular expression of these proteins (Figure 4). ER stressed cells may be preferentially loading EVs with these tetraspanins, while also downregulating their production, resulting in lower intracellular expression.

Previous work has shown that cellular exposure to tunicamycin not only induces ER stress, but can also activate autophagy, therefore we aimed to assess whether autophagy is activated in OLs experiencing tunicamycin-induced ER stress (Ogata et al., 2006). Although we did not observe significant differences in Beclin or ATG-5 expression (Figures 5A, B), we did observe significant upregulation of LC3B expression after ER stress (Figure 5C). We also observed double banding of LC3B, particularly in the ER stressed cells, which indicates the conversion of LC3I (top band) to LC3II (bottom band), which is then targeted to autophagic membranes (Tanida et al., 2008). Tunicamycin-induced ER stress has previously been shown to lead to increased autophagosome formation (Ogata et al., 2006). Interestingly however, our DAPGreen autophagy assay, which functions to detect the formation of autophagosomes and has been reported to have a high correlation with LC3 expression and localization, indicated that autophagosome formation was significantly downregulated in these cells (Figure 5D) (Iwashita et al., 2018). Future work should perform a colocalization study to determine if LC3 is indeed present in the same locations as DAPGreen-detected autophagosomes in these cells. Additionally, very high expression of LC3B in EVs and extracellular proteins released from cells undergoing ER stress (Figure 5E) was also observed. We hypothesize that these data may indicate that LC3B is being released from cells in EVs through the secretory autophagy pathway, LC3-dependent extracellular vesicle loading and secretion (LDELS) (Solvik et al., 2022; Leidal and Jayanta, 2020). One caveat to this is that our EV western blots were normalized to total volume (15 µL) instead of total protein so this effect may be due, in part, to more vesicles being present in the tunicamycin exposed samples relative to the control (Figure 3B) however, the difference in total EV concentration was not statistically significant between the two sample types.

Because ER stress can halt protein production, we wanted to assess whether expression of several key myelination proteins, MBP, PLP, and CNPase, would be negatively impacted. For all three proteins, we observed significant downregulation after ER stress (Figure 6). Interestingly we did not observe any significant differences in expression of MBP at the gene level (Figure 7A). PLP is the most abundant myelination protein in the CNS, while MBP is the second-most abundant (Martinsen and Kursula, 2022; Greer et al., 2020). Although reduced expression of these proteins in MS may be partially attributed to autoimmune dysfunction, targeting them as autoantigens, it is possible that ER stress, which is observed in MS, may contribute to its altered expression (Martinsen and Kursula, 2022; Lin et al., 2008; Stone and Lin, 2015; Greer et al., 2020). CNPase is the first myelination specific protein formed in OLs and plays a significant role in the formation of the myelin sheath where it comprises 4% of total protein (Angelis and Braun, 1994). It is a membrane bound protein that also serves as a microtubule-associated protein (MAP) to facilitate microtubule formation, so it has a likely role in the formation of the myelin cytoskeleton (Bifulco et al., 2002; Laezza et al., 1997). It has also been shown to associate with mitochondria and may play important roles in modulating cellular survival and death mechanisms (Olga et al., 2020). Although CNPase mRNA was not statistically significantly upregulated after ER stress, there was a trend of increased expression that almost reached significance (*p* = 0.0577) (Figure 7B). Since this protein plays such a pivotal role in the formation and organization of myelin sheaths and cell viability, it is possible that the cell is compensating for its reduced availability at the protein level by increasing its expression at the gene level. Further, we also saw increased expression of ERBB4 mRNA after ER stress (Figure 7D). The ErbB receptor plays an important role in remyelination, therefore we hypothesize that ERBB4 mRNA is upregulated in response to the significant loss of key myelination proteins that occurs during ER stress (Bartus et al., 2019). We also probed EV and protein fractions for expression of MBP, PLP, and CNPase proteins, but did not observe their presence in these samples (Data not shown).

Lastly, because some of miR-29a-3p′s predicted binding targets include PLP and myelin oligodendrocyte glycoprotein (Targetscan.org) and has been associated with autophagy, we assessed its expression in cells and their released EVs and proteins. Although no statistically significant changes were observed in the cell lysates or protein fractions, we did observe a trend of increased miR-29a-3p expression (Figures 8A, C). There was a significant increase in its expression in EVs released from ER stressed cells (Figure 8B). Interestingly, previous research has shown that peripheral myelinating cells (Schwann cells) upregulate the expression of miR-29a-3p after injury which contributes to functional recovery (Shen et al., 2022). It is plausible that OLs are employing a similar mechanism to upregulate miR-29a-3p for recovery from injury associated with ER stress. Cells undergoing ER stress may release EVs containing miR-29a-3p as a mechanism to protect neighboring cells. Further, in pulmonary endothelial cells, it was found that miR-29a-3p activated autophagy (Li et al., 2023). We observed strong LC3B banding in EVs released from ER stressed cells (Figure 5E) with concurrent overexpression of miR-29a-3p (Figure 8B), which may indicate that ER stressed cells are releasing EVs geared towards protecting their neighboring cells by transferring miR-29a-3p and LC3B to activate autophagy preemptively since the parent cells are experiencing ER stress and subsequent accumulation of misfolded protein aggregates.

Overall, this study suggests that OLs experiencing ER stress significantly reduce their ability to produce key myelination proteins, while simultaneously employ mechanisms to protect themselves (increased expression of ERBB4), and their neighbors through the production and release of EVs carrying miR-29a-3p and LC3B. These EVs may be important biomarkers for early indications of OL pathologies in the context of MS and ER stress and may be important targets for future therapeutics.

## Data Availability

The raw data supporting the conclusions of this article will be made available by the authors, without undue reservation.
