# Supplementary material for: Endoplasmic reticulum stress alters myelin associated protein expression and extracellular vesicle composition in human oligodendrocytes
Source: Front Mol Biosci. 2024 Oct 1;11:1432945. doi: 10.3389/fmolb.2024.1432945 (PMC11473301; doi:10.3389/fmolb.2024.1432945)
Supplement: Supplementary file 1 [file Presentation1.PPTX]

## Slide 1
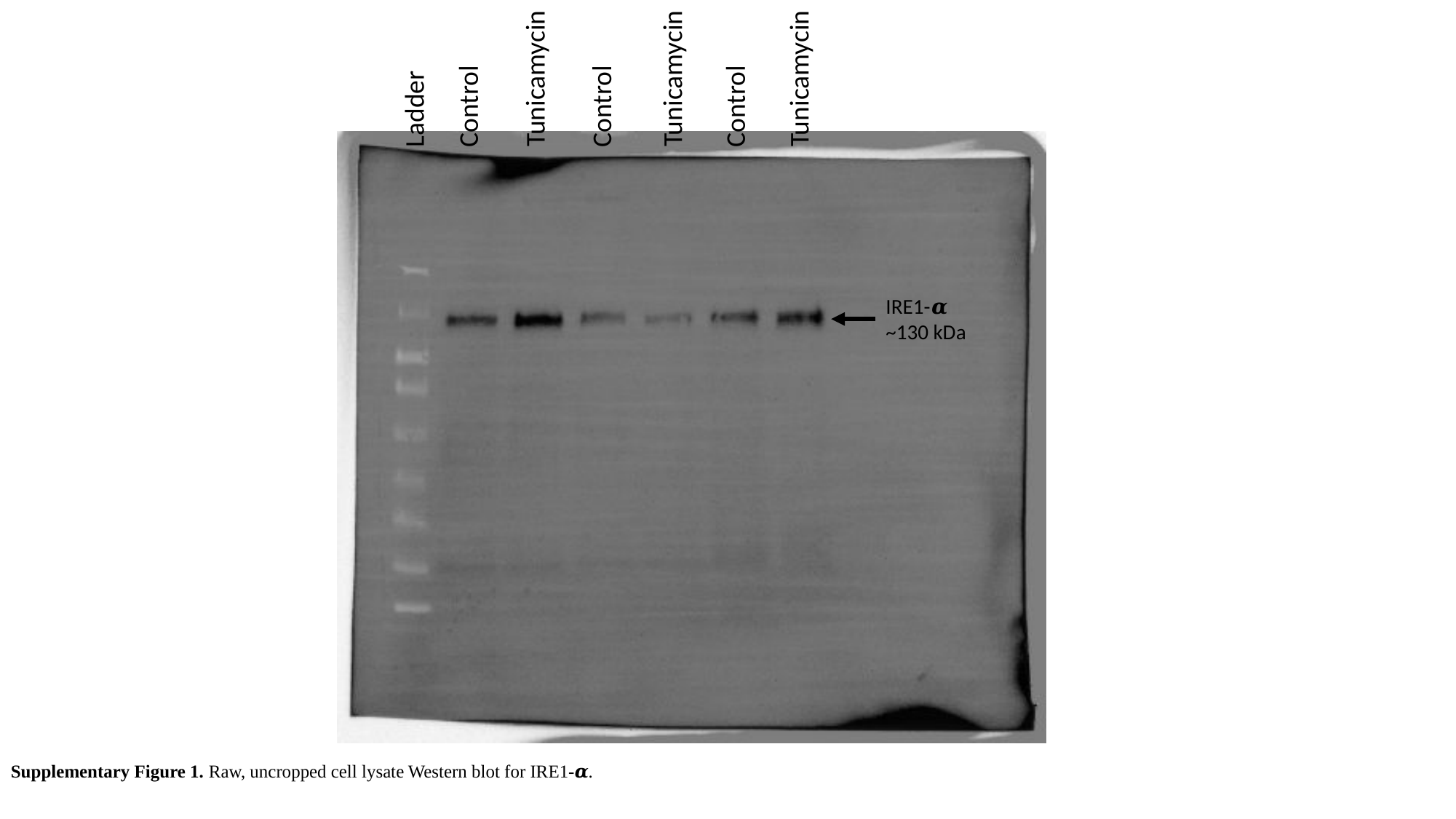

Tunicamycin
Tunicamycin
Tunicamycin
Control
Control
Control
Ladder
IRE1-𝜶
~130 kDa
Supplementary Figure 1. Raw, uncropped cell lysate Western blot for IRE1-𝜶.

## Slide 2
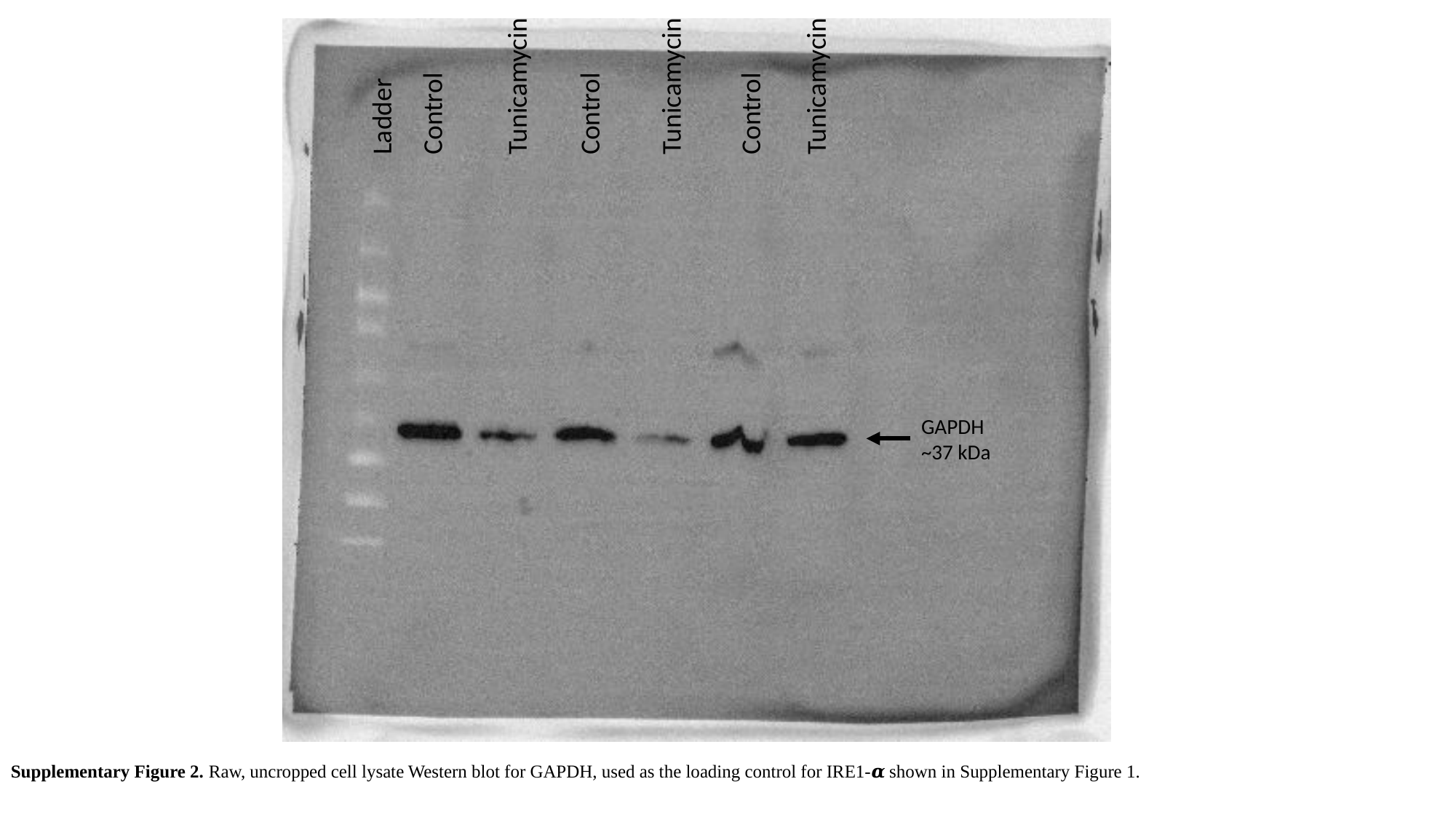

Tunicamycin
Tunicamycin
Tunicamycin
Control
Control
Control
Ladder
GAPDH
~37 kDa
Supplementary Figure 2. Raw, uncropped cell lysate Western blot for GAPDH, used as the loading control for IRE1-𝜶 shown in Supplementary Figure 1.

## Slide 3
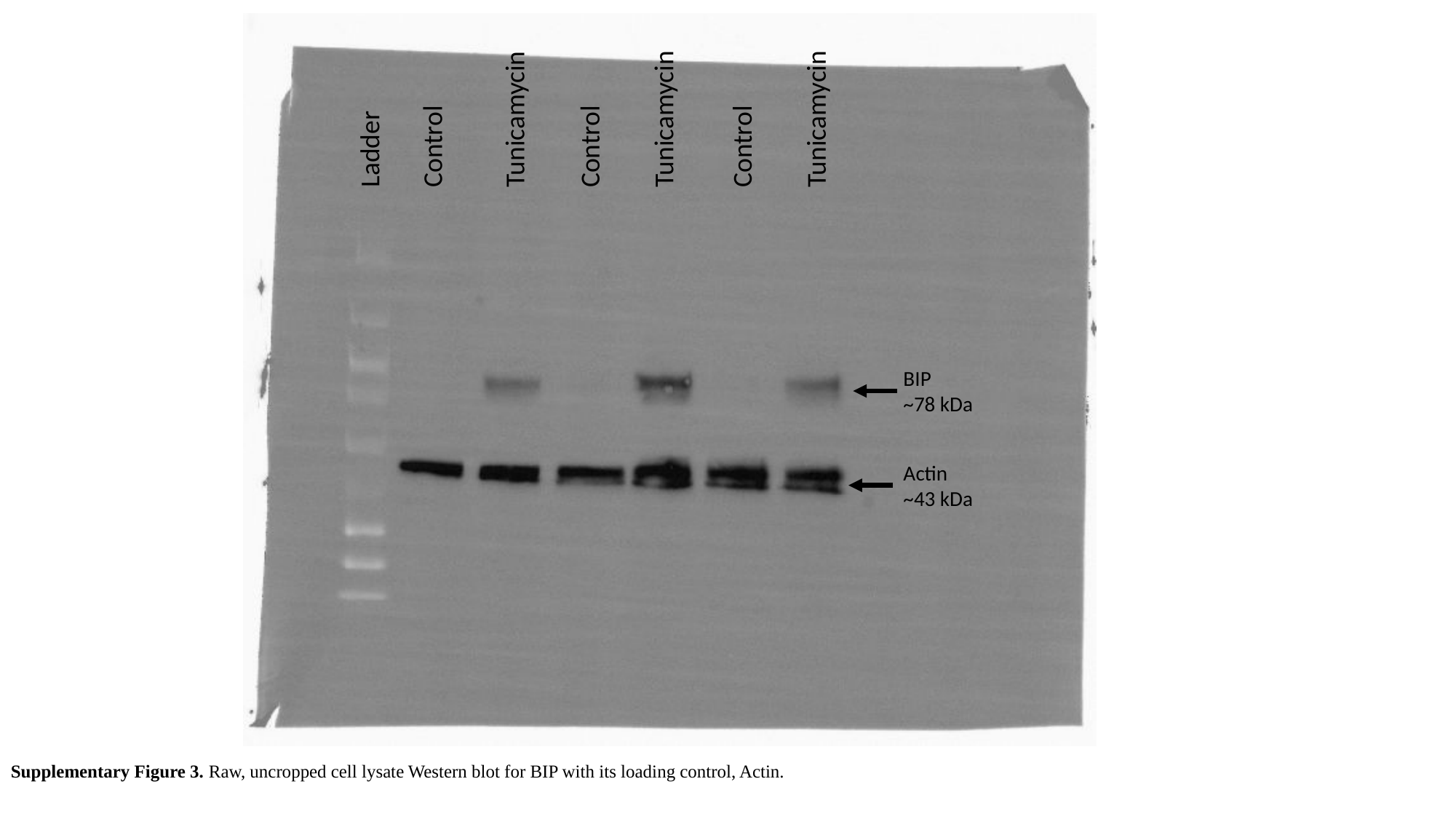

Tunicamycin
Tunicamycin
Tunicamycin
Control
Control
Control
Ladder
BIP
~78 kDa
Actin
~43 kDa
Supplementary Figure 3. Raw, uncropped cell lysate Western blot for BIP with its loading control, Actin.

## Slide 4
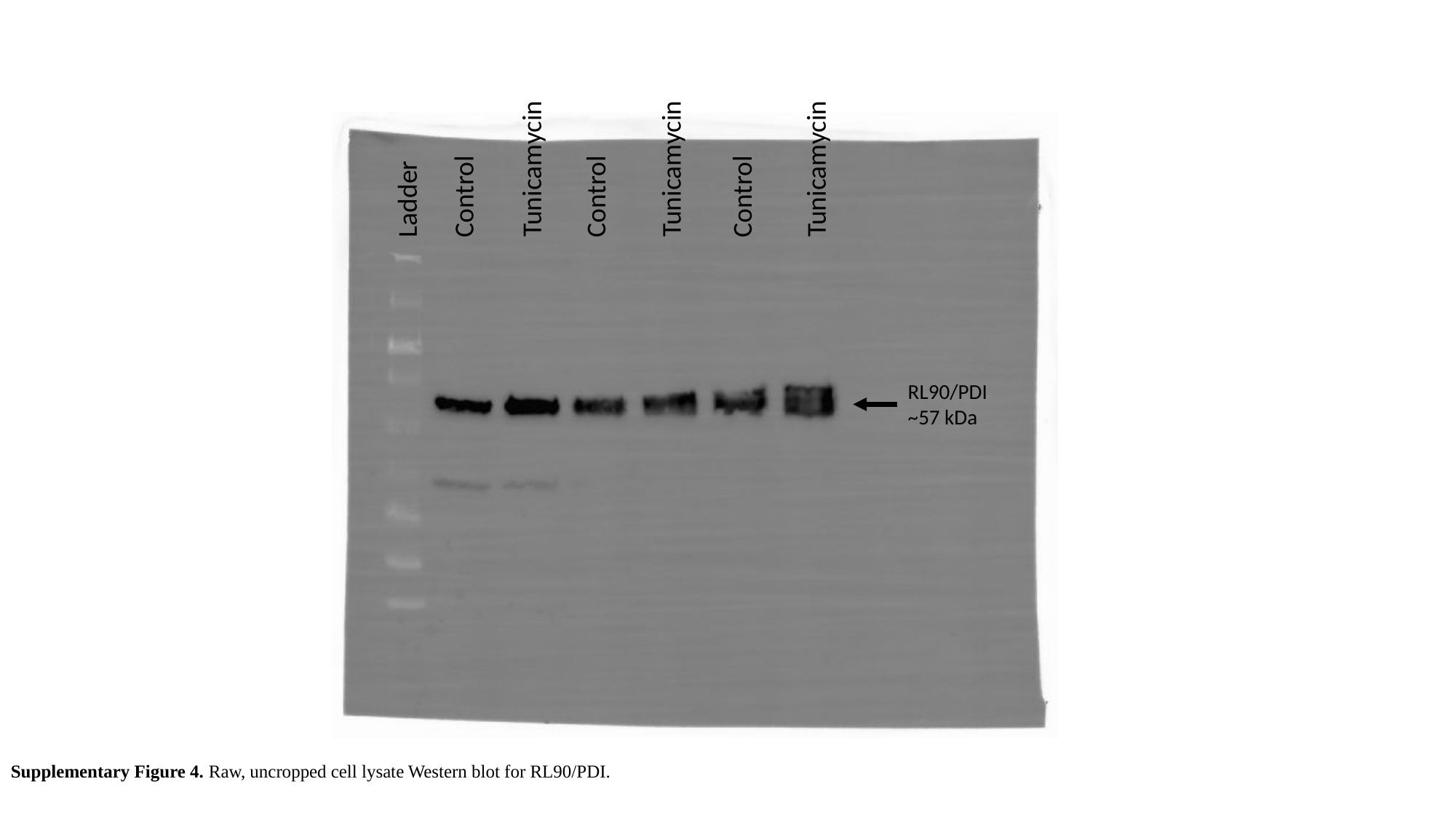

Tunicamycin
Tunicamycin
Tunicamycin
Control
Control
Control
Ladder
RL90/PDI
~57 kDa
Supplementary Figure 4. Raw, uncropped cell lysate Western blot for RL90/PDI.

## Slide 5
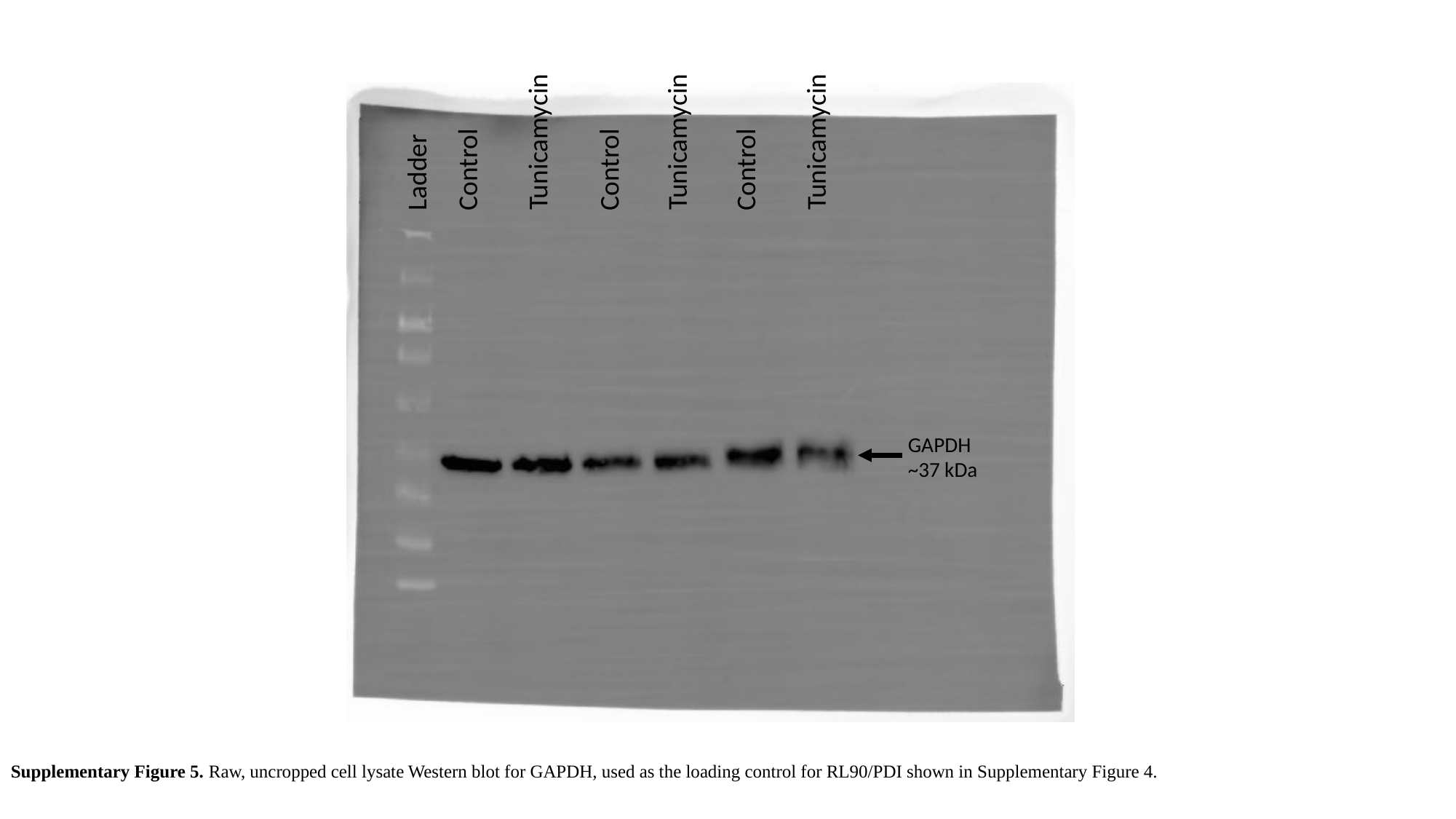

Tunicamycin
Tunicamycin
Tunicamycin
Control
Control
Control
Ladder
GAPDH
~37 kDa
Supplementary Figure 5. Raw, uncropped cell lysate Western blot for GAPDH, used as the loading control for RL90/PDI shown in Supplementary Figure 4.

## Slide 6
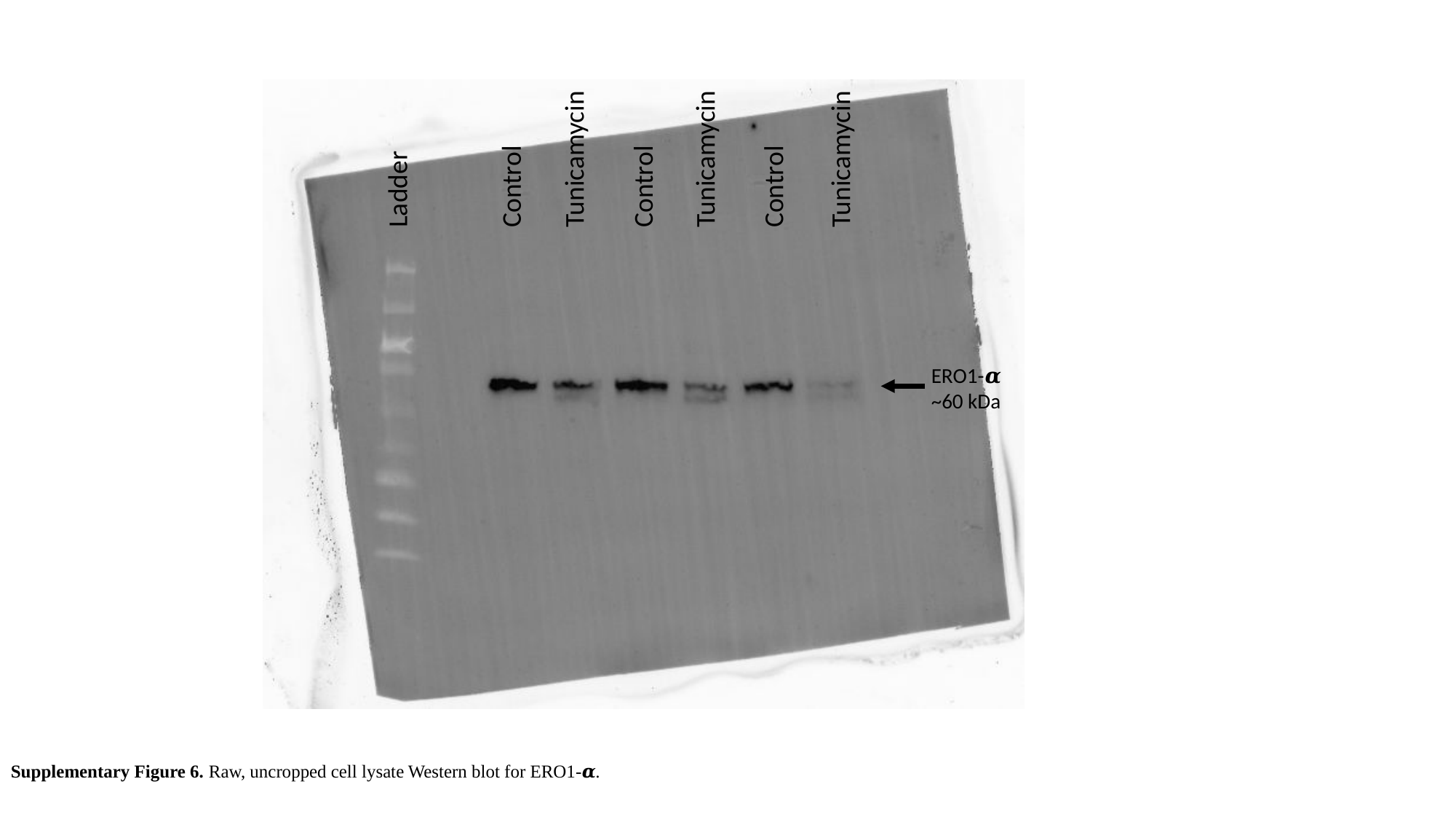

Tunicamycin
Tunicamycin
Tunicamycin
Control
Control
Control
Ladder
ERO1-𝜶
~60 kDa
Supplementary Figure 6. Raw, uncropped cell lysate Western blot for ERO1-𝜶.

## Slide 7
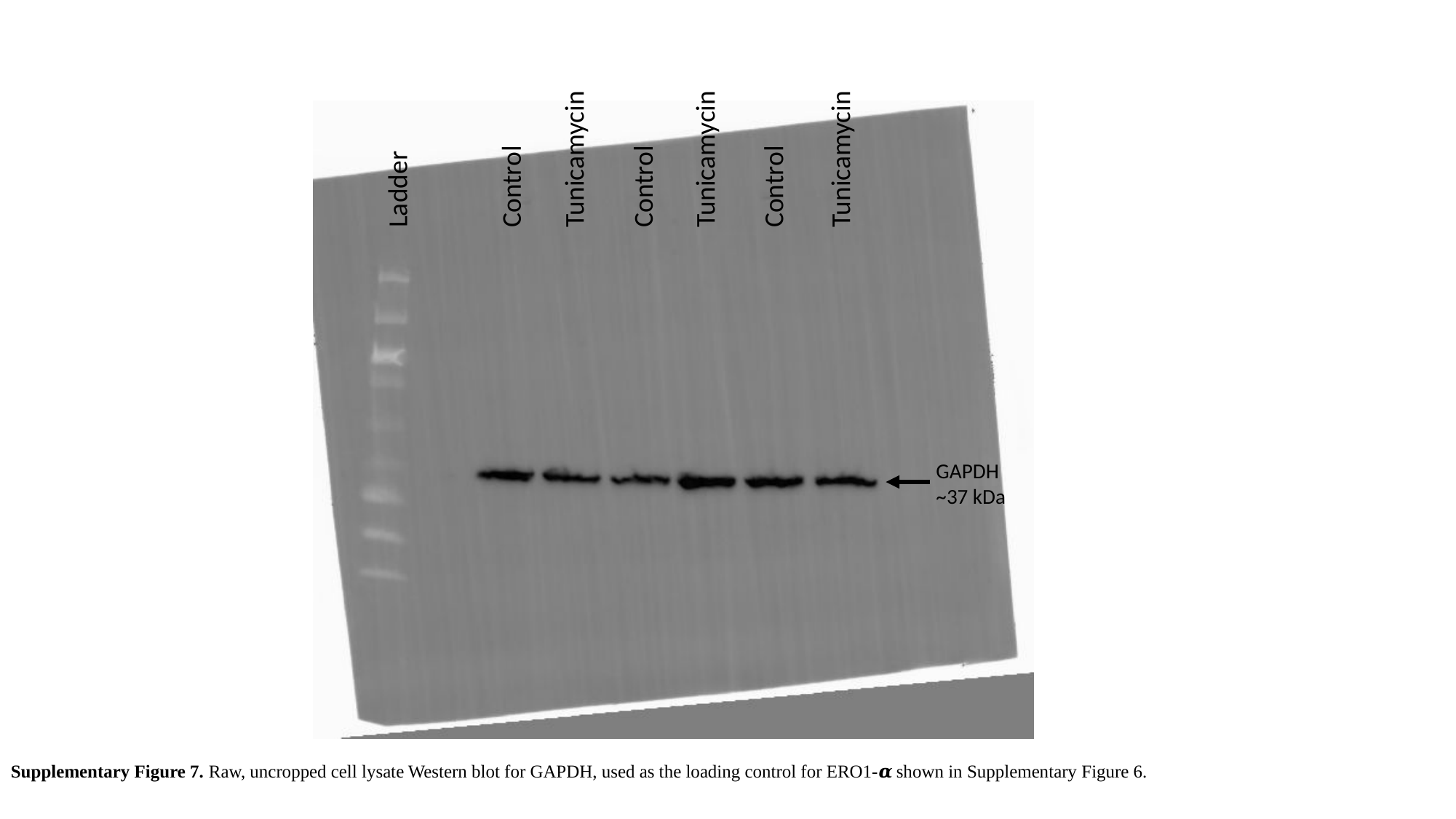

Tunicamycin
Tunicamycin
Tunicamycin
Control
Control
Control
Ladder
GAPDH
~37 kDa
Supplementary Figure 7. Raw, uncropped cell lysate Western blot for GAPDH, used as the loading control for ERO1-𝜶 shown in Supplementary Figure 6.

## Slide 8
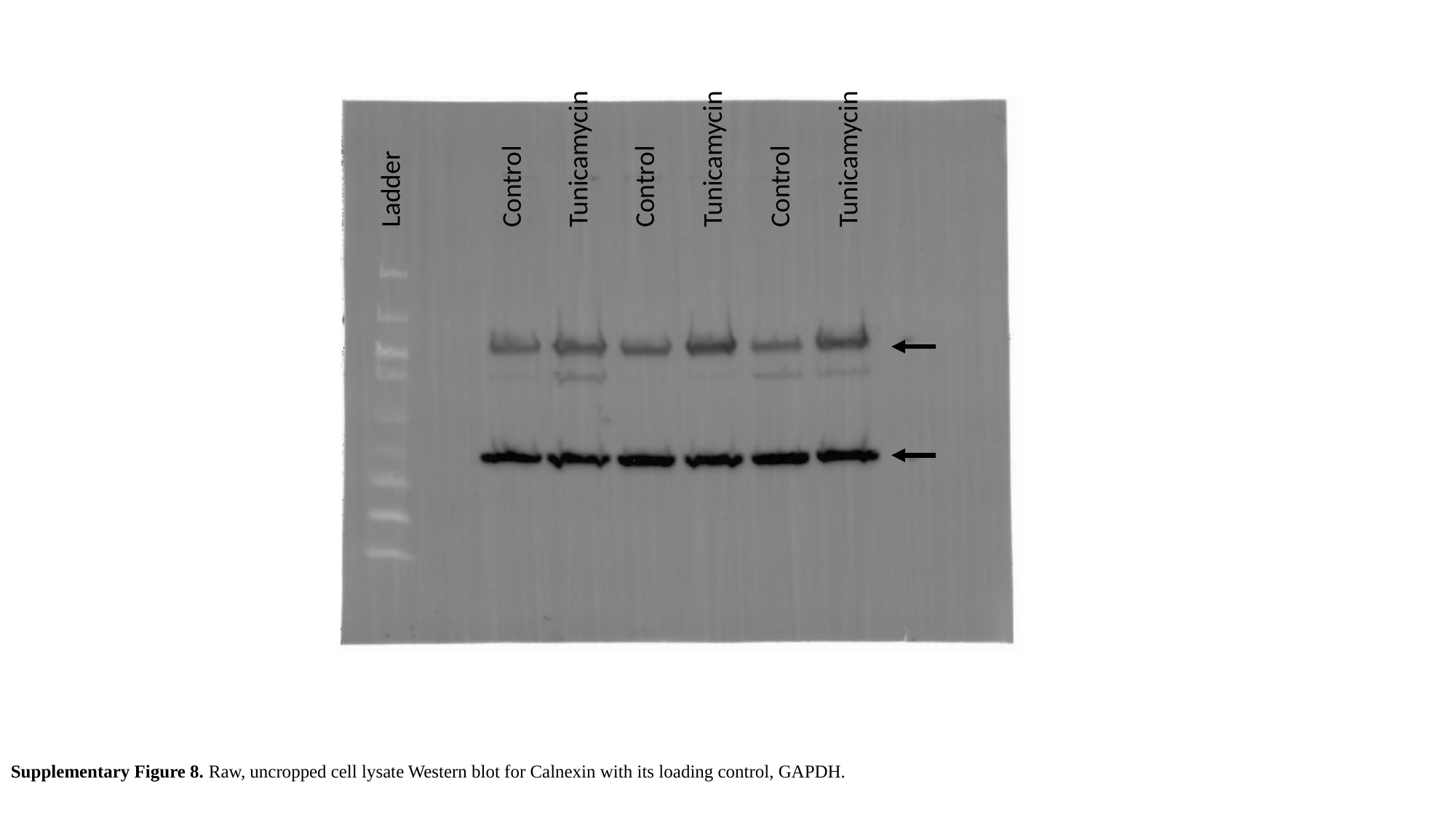

Tunicamycin
Tunicamycin
Tunicamycin
Control
Control
Control
Ladder
Calnexin
~90 kDa
GAPDH
~37 kDa
Supplementary Figure 8. Raw, uncropped cell lysate Western blot for Calnexin with its loading control, GAPDH.

## Slide 9
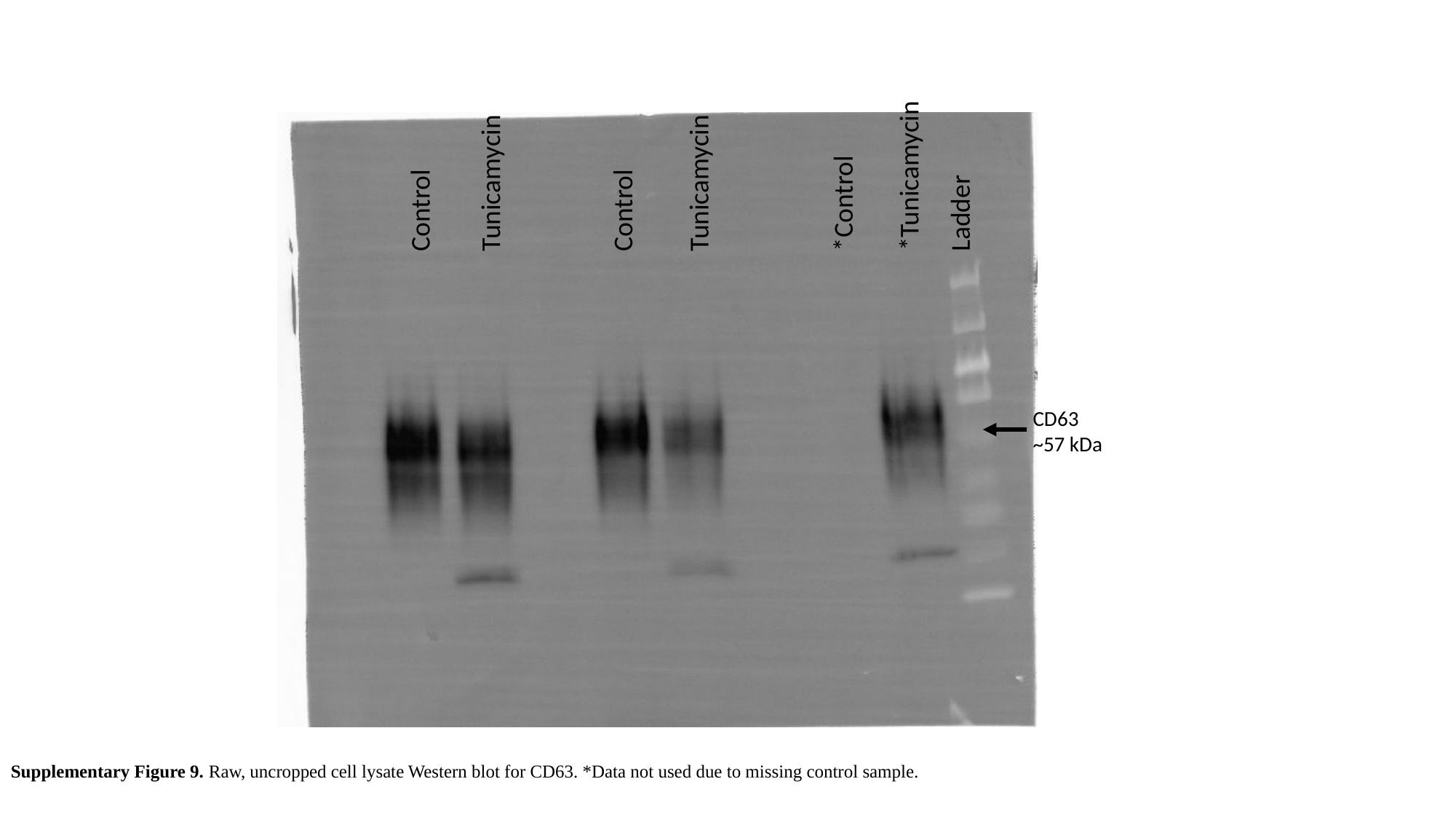

*Tunicamycin
Tunicamycin
Tunicamycin
*Control
Control
Control
Ladder
CD63
~57 kDa
Supplementary Figure 9. Raw, uncropped cell lysate Western blot for CD63. *Data not used due to missing control sample.

## Slide 10
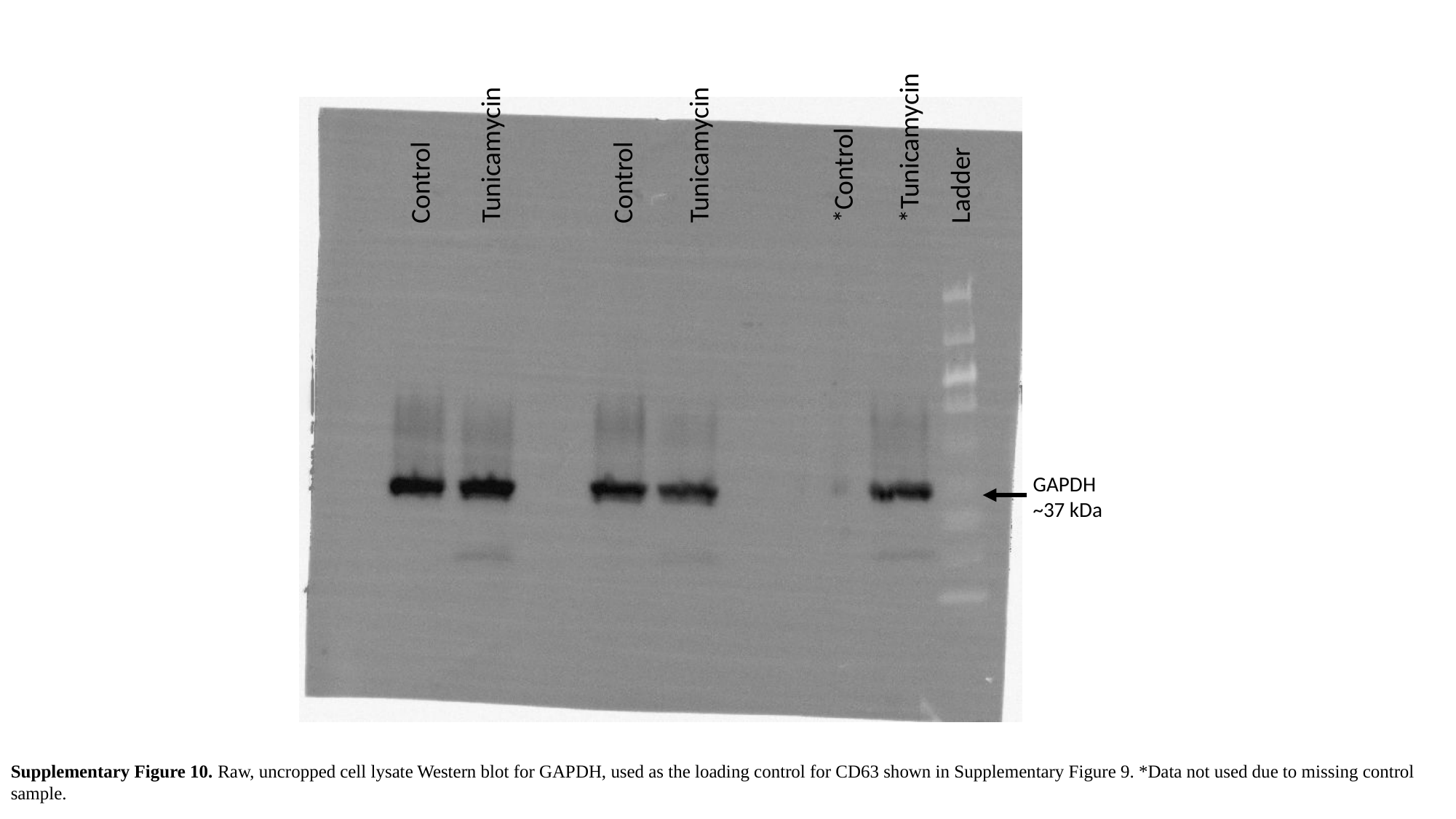

*Tunicamycin
Tunicamycin
Tunicamycin
*Control
Control
Control
Ladder
GAPDH
~37 kDa
Supplementary Figure 10. Raw, uncropped cell lysate Western blot for GAPDH, used as the loading control for CD63 shown in Supplementary Figure 9. *Data not used due to missing control sample.

## Slide 11
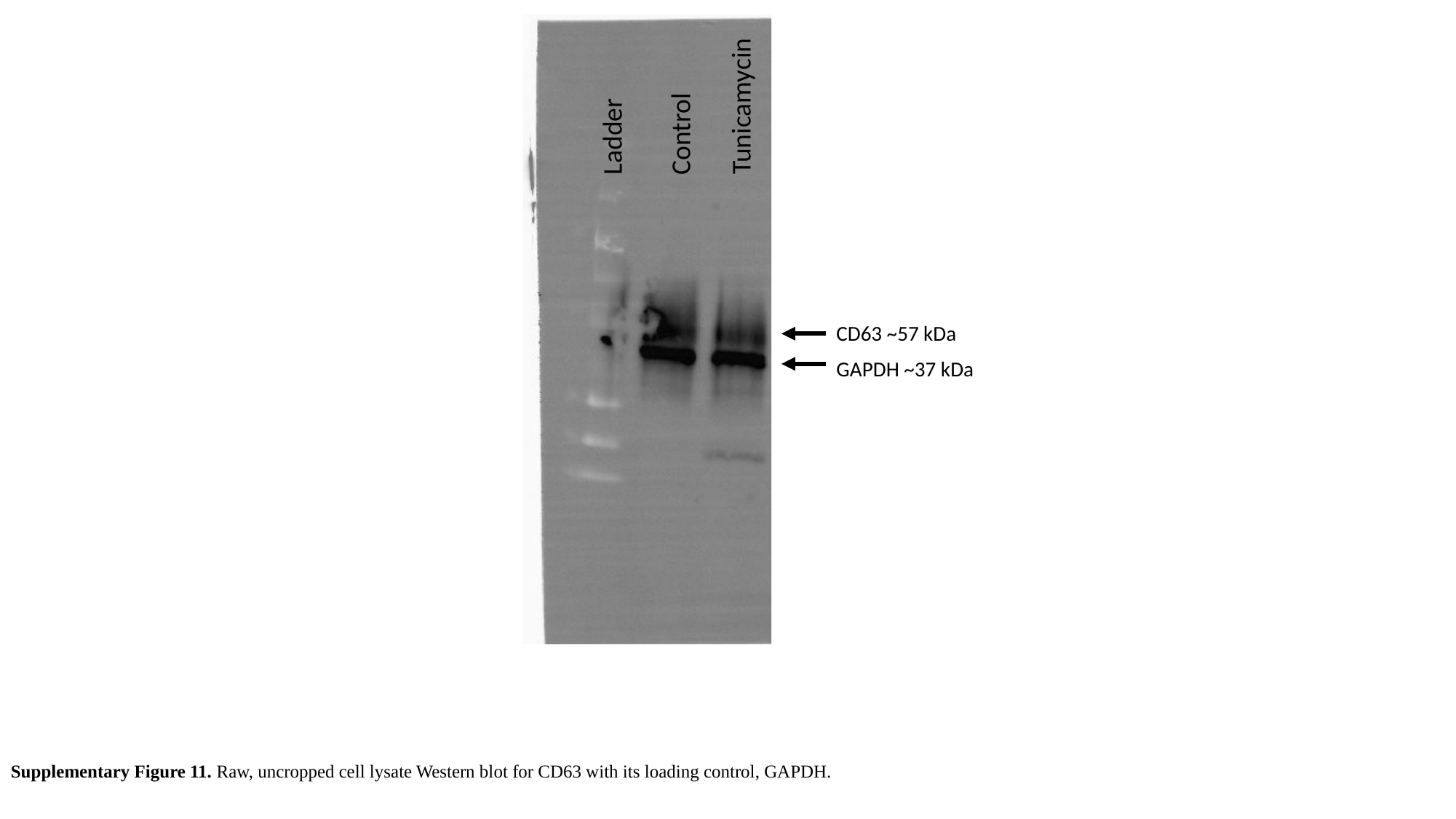

Tunicamycin
Control
Ladder
CD63 ~57 kDa
GAPDH ~37 kDa
Supplementary Figure 11. Raw, uncropped cell lysate Western blot for CD63 with its loading control, GAPDH.

## Slide 12
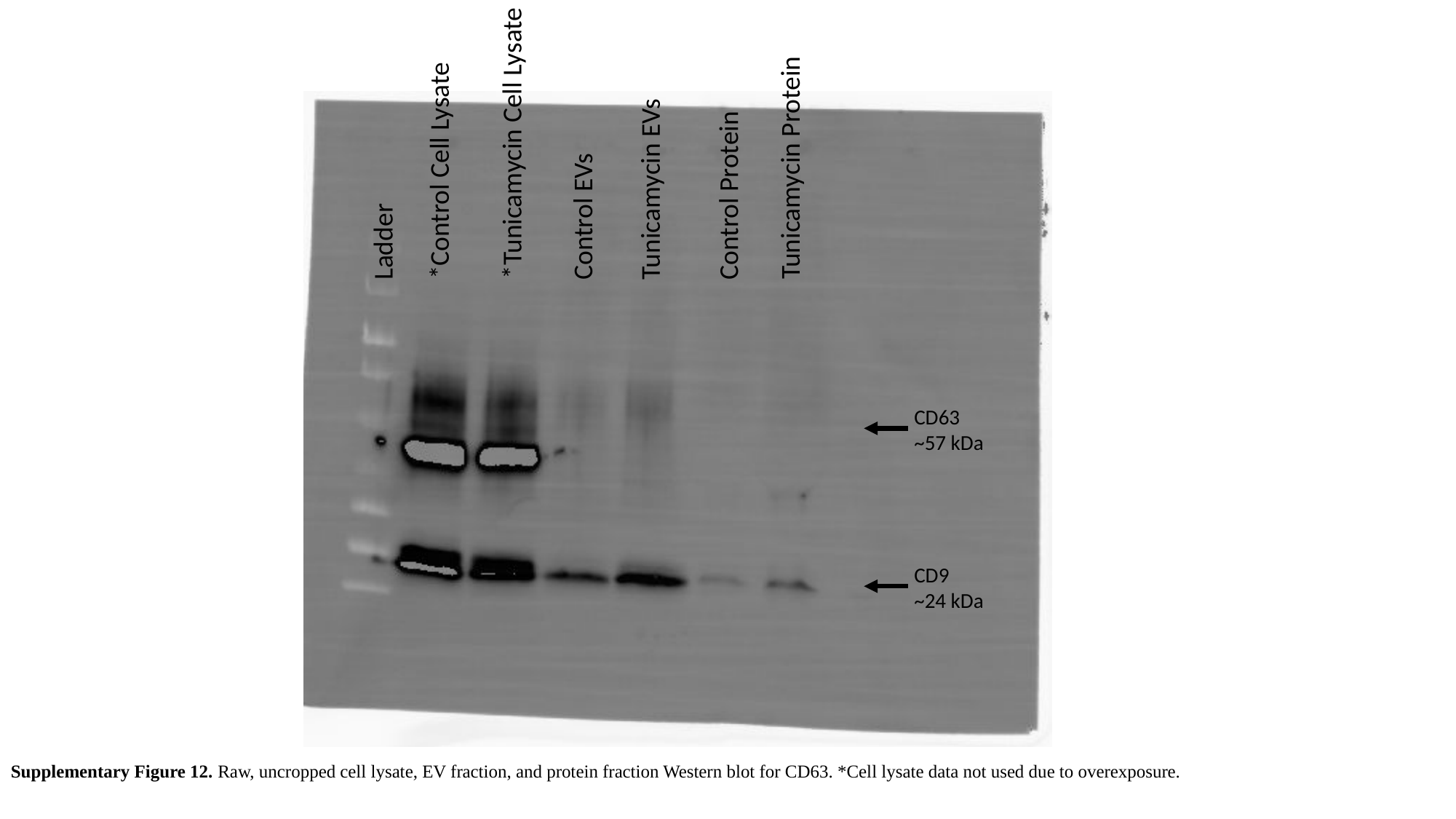

*Tunicamycin Cell Lysate
Tunicamycin Protein
*Control Cell Lysate
Tunicamycin EVs
Control Protein
Control EVs
Ladder
CD63
~57 kDa
CD9
~24 kDa
Supplementary Figure 12. Raw, uncropped cell lysate, EV fraction, and protein fraction Western blot for CD63. *Cell lysate data not used due to overexposure.

## Slide 13
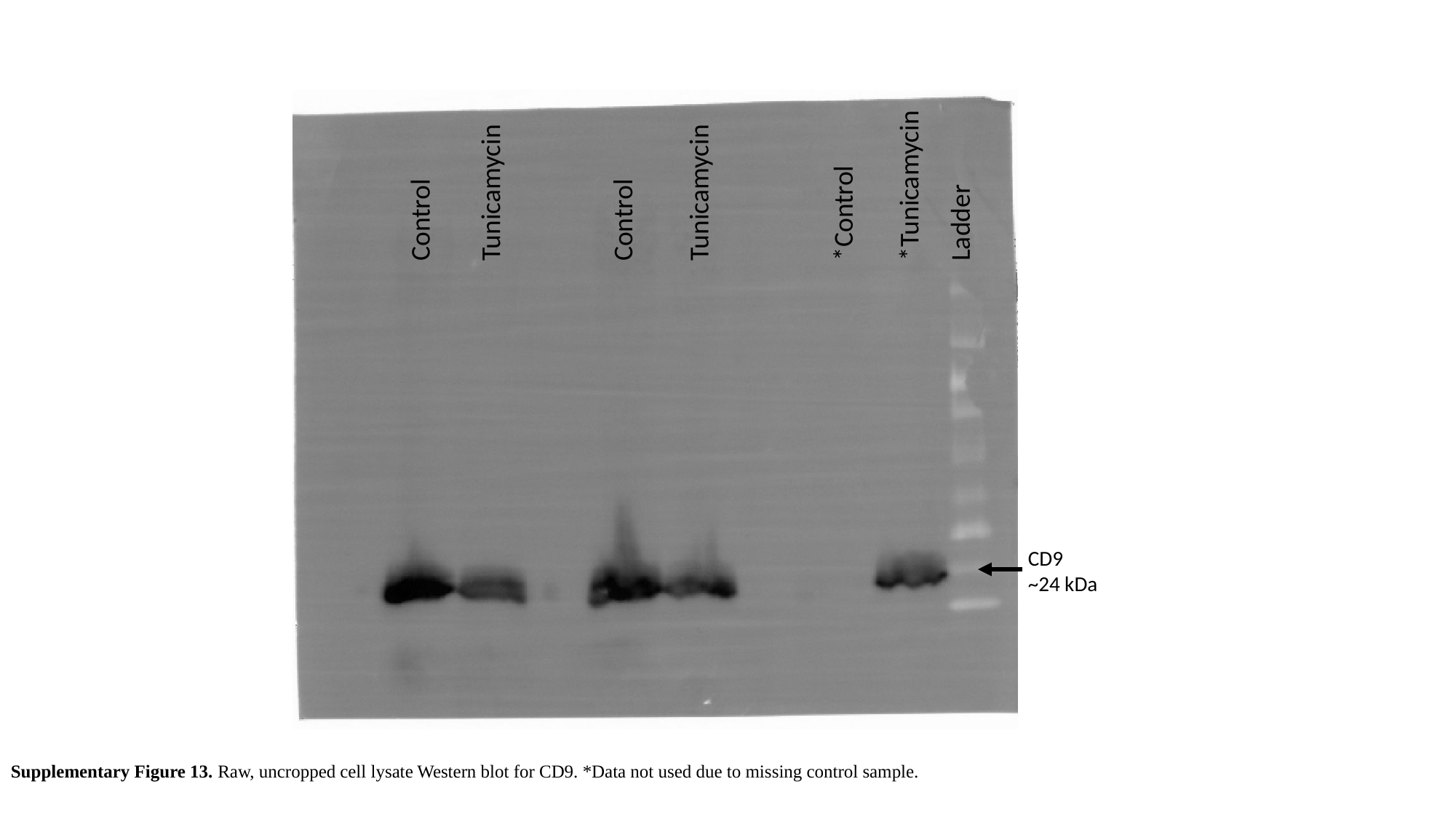

*Tunicamycin
Tunicamycin
Tunicamycin
*Control
Control
Control
Ladder
CD9
~24 kDa
Supplementary Figure 13. Raw, uncropped cell lysate Western blot for CD9. *Data not used due to missing control sample.

## Slide 14
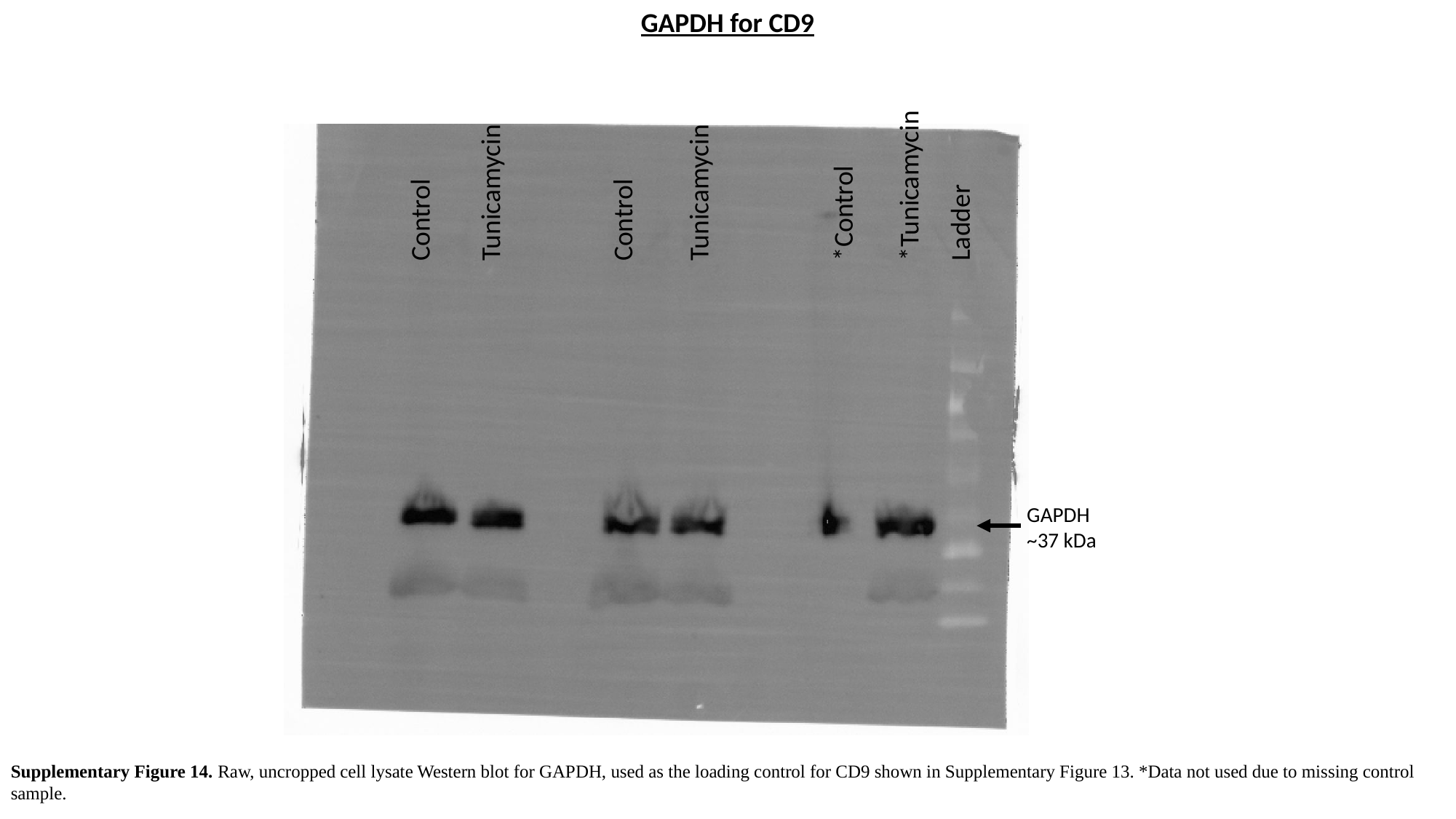

GAPDH for CD9
*Tunicamycin
Tunicamycin
Tunicamycin
*Control
Control
Control
Ladder
GAPDH
~37 kDa
Supplementary Figure 14. Raw, uncropped cell lysate Western blot for GAPDH, used as the loading control for CD9 shown in Supplementary Figure 13. *Data not used due to missing control sample.

## Slide 15
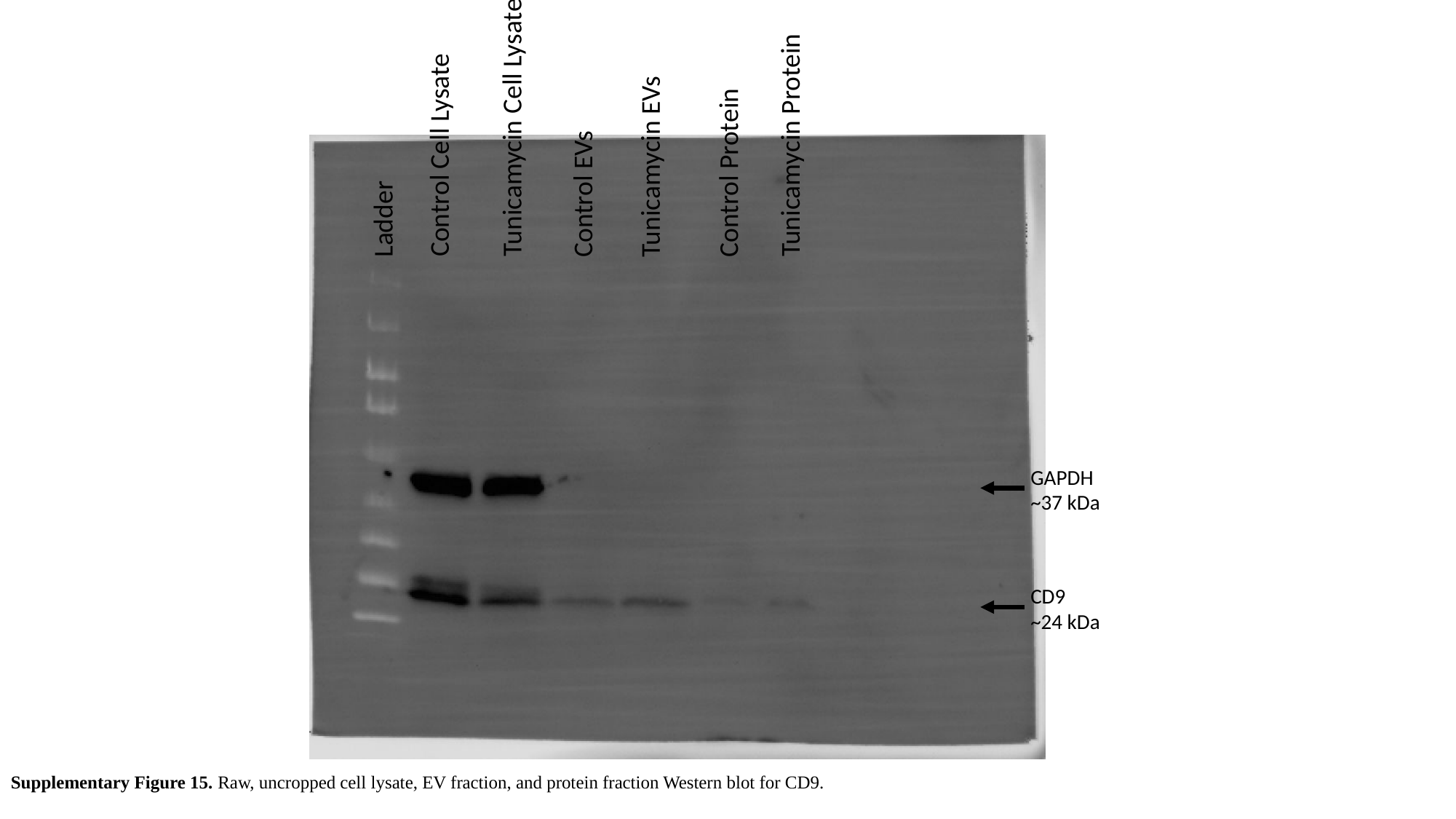

Tunicamycin Cell Lysate
Tunicamycin Protein
Control Cell Lysate
Tunicamycin EVs
Control Protein
Control EVs
Ladder
GAPDH
~37 kDa
CD9
~24 kDa
Supplementary Figure 15. Raw, uncropped cell lysate, EV fraction, and protein fraction Western blot for CD9.

## Slide 16
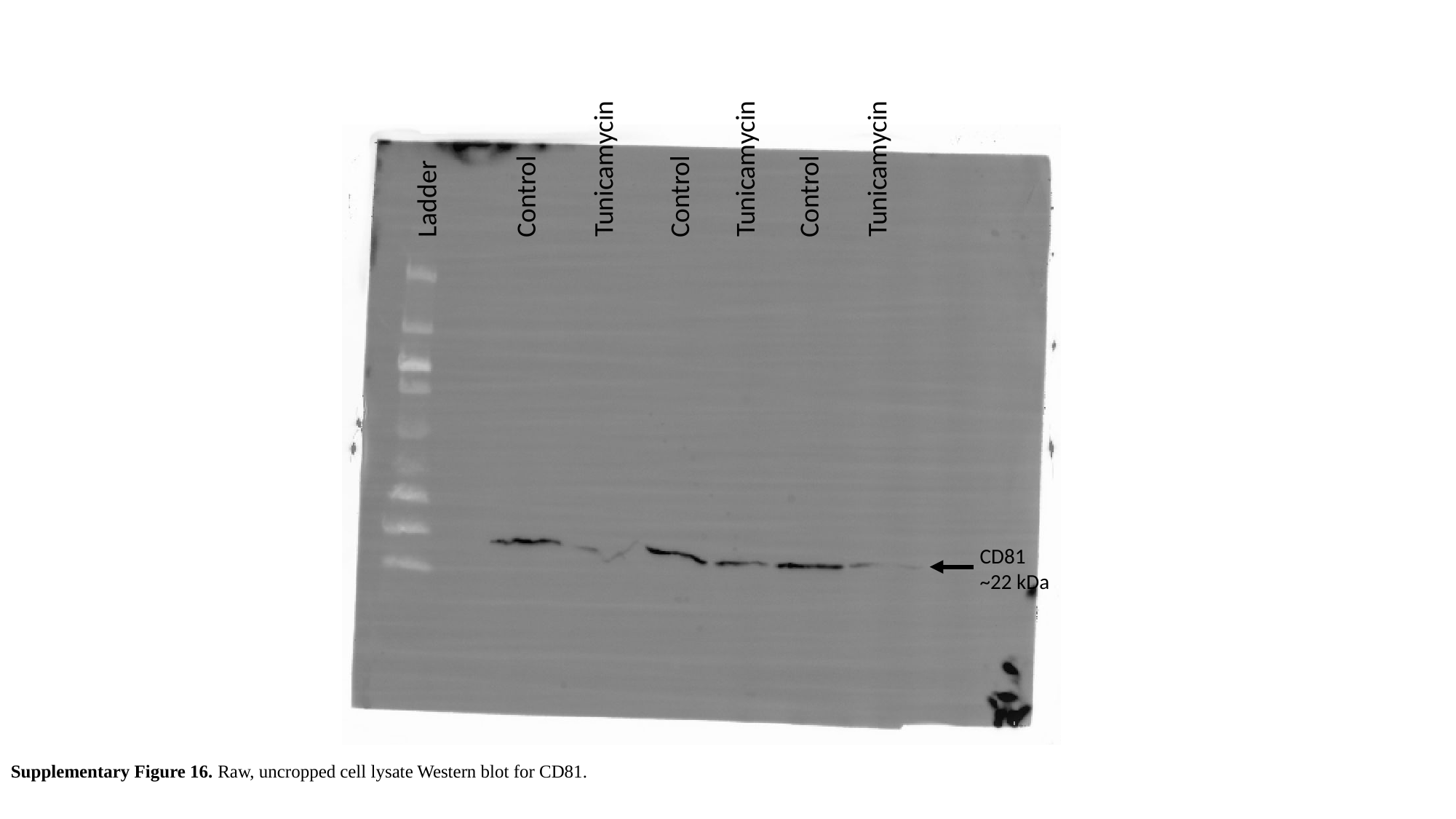

Tunicamycin
Tunicamycin
Tunicamycin
Control
Control
Control
Ladder
CD81
~22 kDa
Supplementary Figure 16. Raw, uncropped cell lysate Western blot for CD81.

## Slide 17
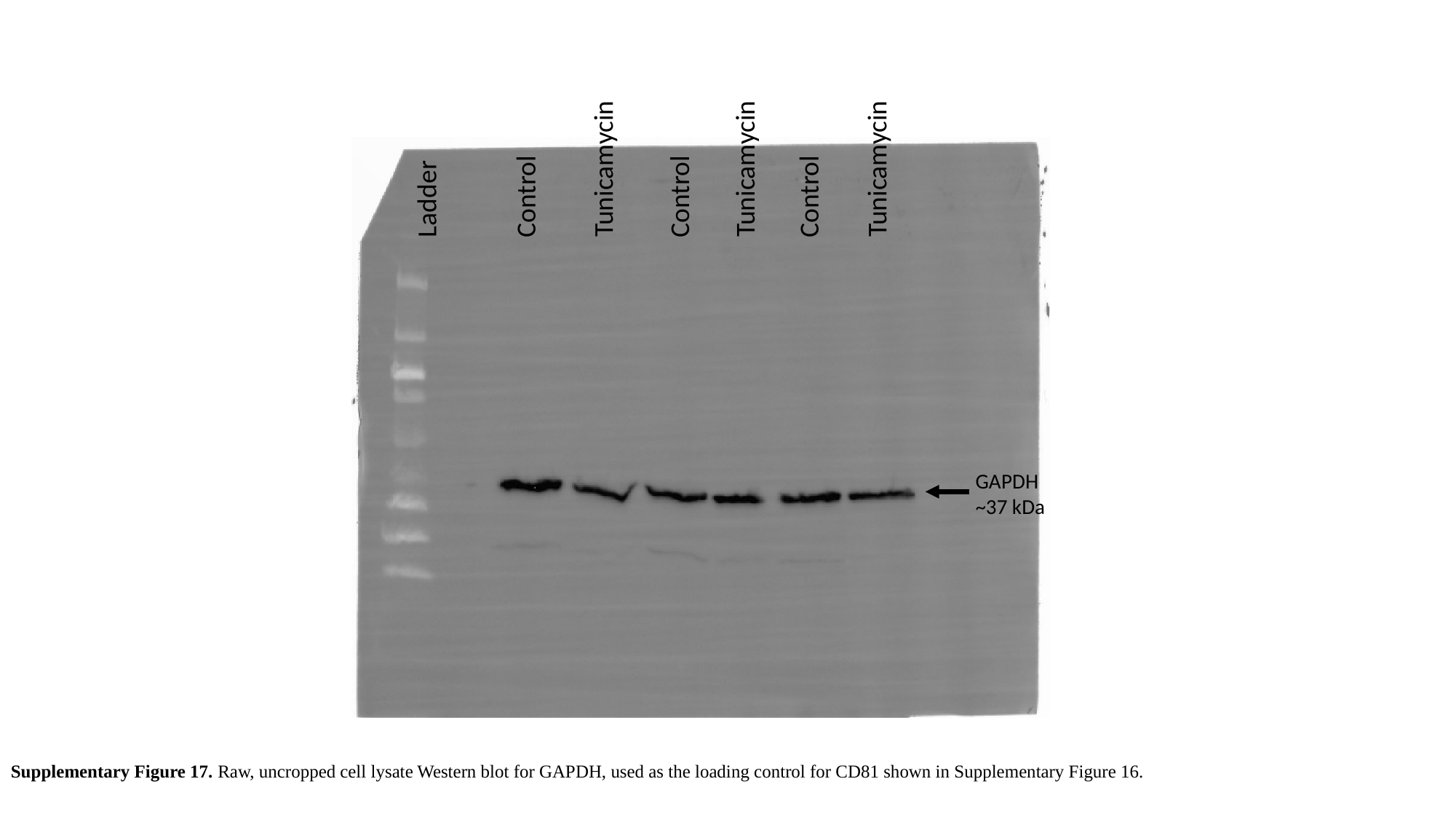

Tunicamycin
Tunicamycin
Tunicamycin
Control
Control
Control
Ladder
GAPDH
~37 kDa
Supplementary Figure 17. Raw, uncropped cell lysate Western blot for GAPDH, used as the loading control for CD81 shown in Supplementary Figure 16.

## Slide 18
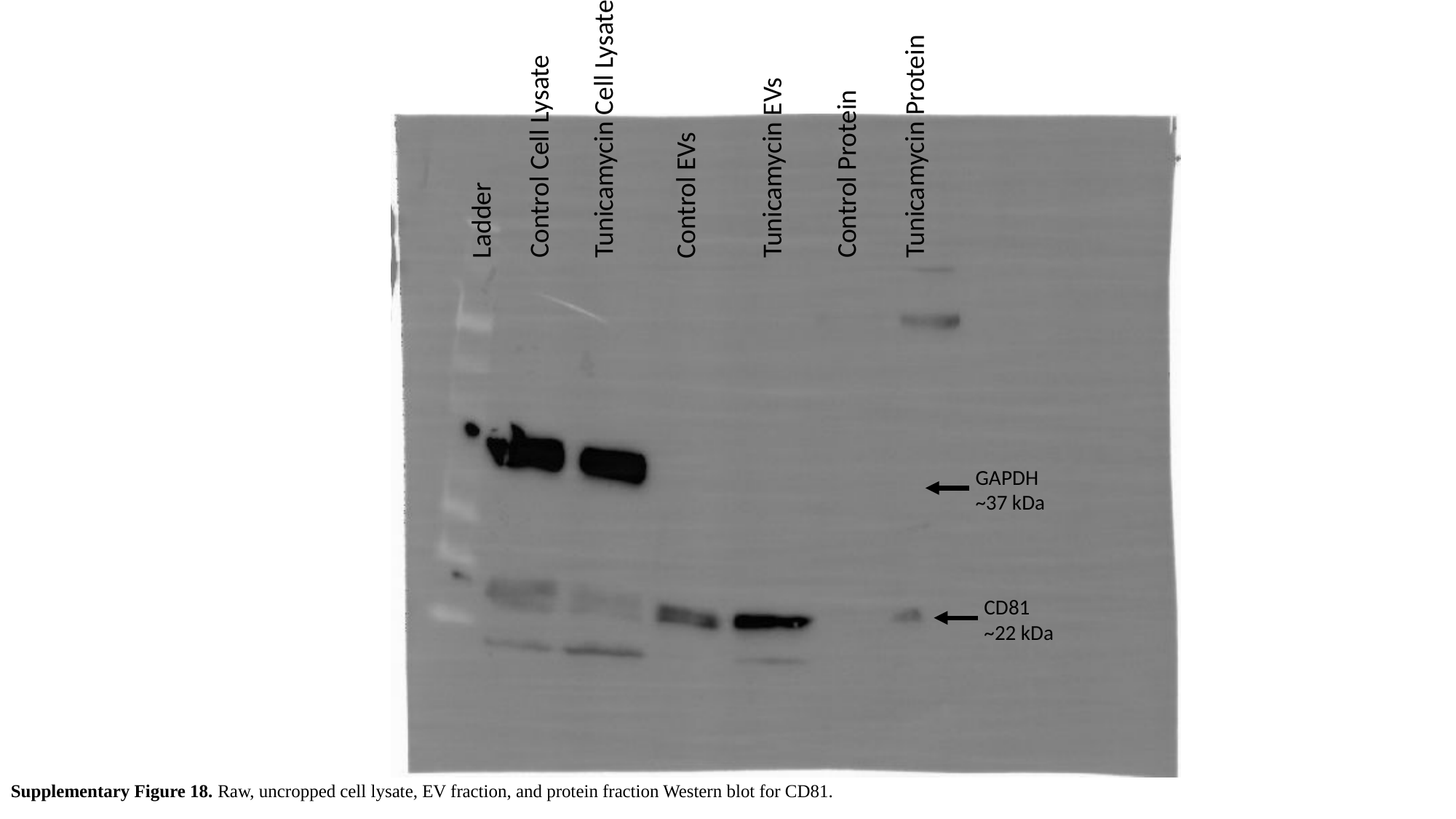

Tunicamycin Cell Lysate
Tunicamycin Protein
Control Cell Lysate
Tunicamycin EVs
Control Protein
Control EVs
Ladder
GAPDH
~37 kDa
CD81
~22 kDa
Supplementary Figure 18. Raw, uncropped cell lysate, EV fraction, and protein fraction Western blot for CD81.

## Slide 19
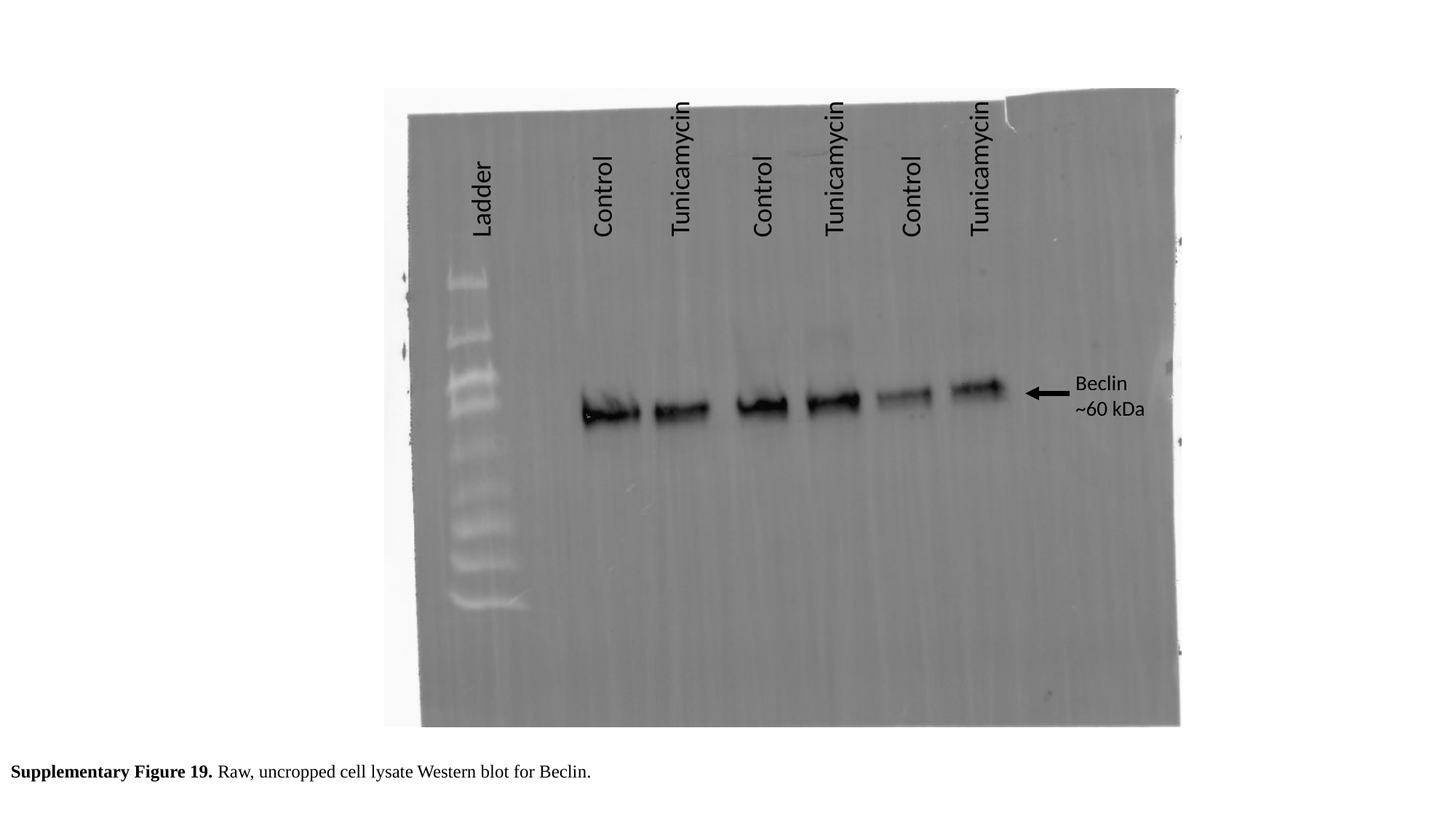

Tunicamycin
Tunicamycin
Tunicamycin
Control
Control
Control
Ladder
Beclin
~60 kDa
Supplementary Figure 19. Raw, uncropped cell lysate Western blot for Beclin.

## Slide 20
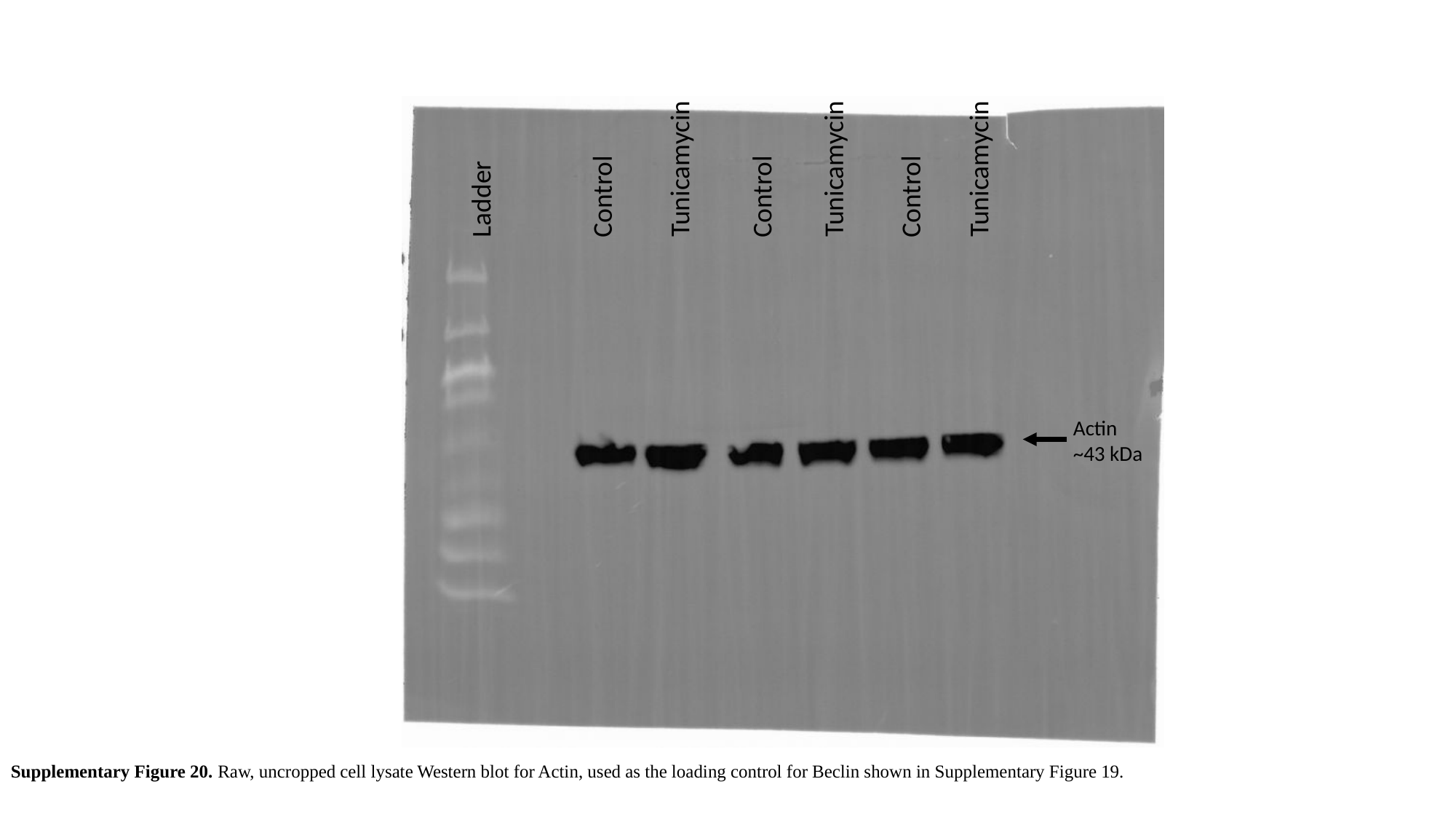

Tunicamycin
Tunicamycin
Tunicamycin
Control
Control
Control
Ladder
Actin
~43 kDa
Supplementary Figure 20. Raw, uncropped cell lysate Western blot for Actin, used as the loading control for Beclin shown in Supplementary Figure 19.

## Slide 21
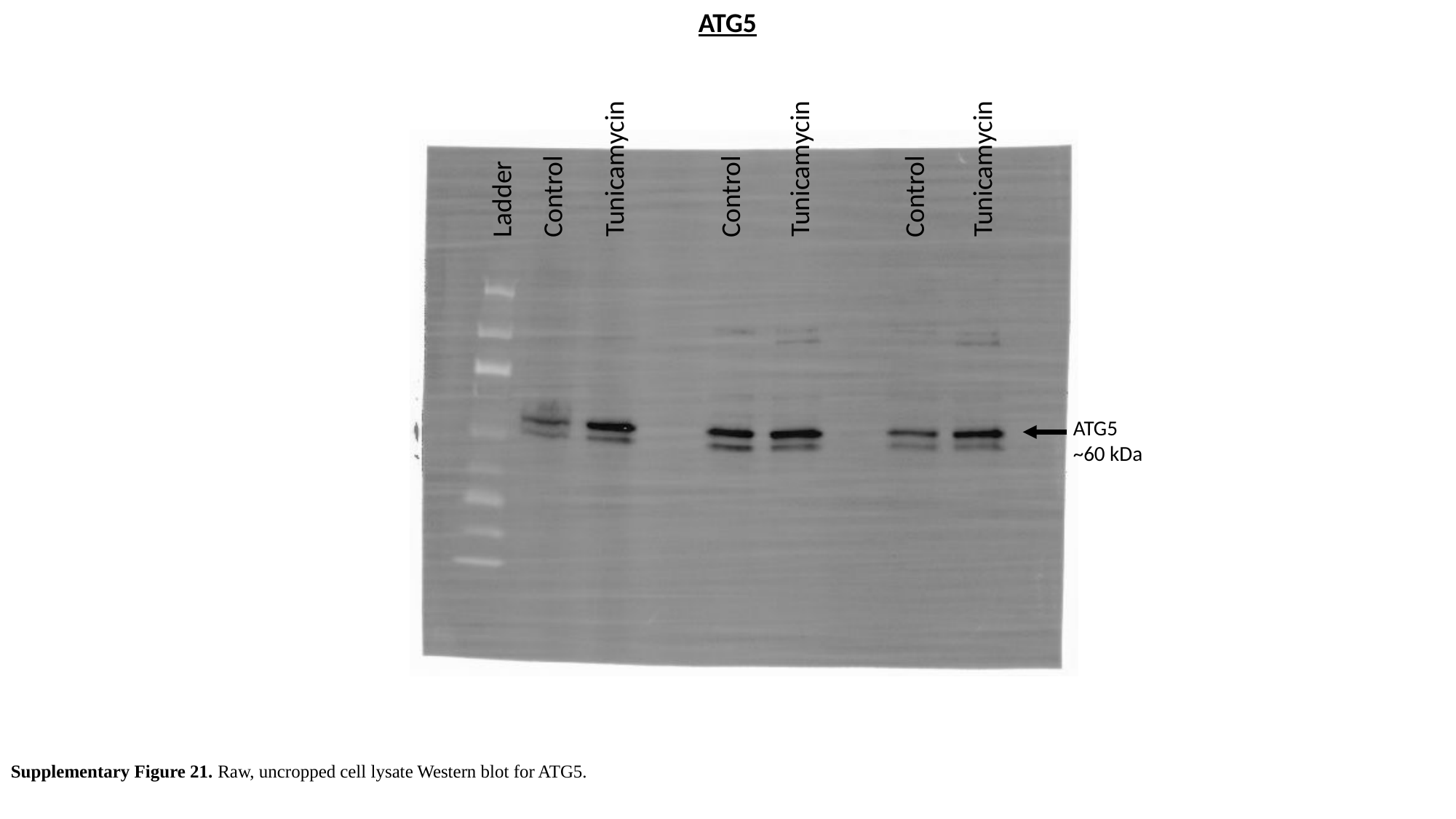

ATG5
Tunicamycin
Tunicamycin
Tunicamycin
Control
Control
Control
Ladder
ATG5
~60 kDa
Supplementary Figure 21. Raw, uncropped cell lysate Western blot for ATG5.

## Slide 22
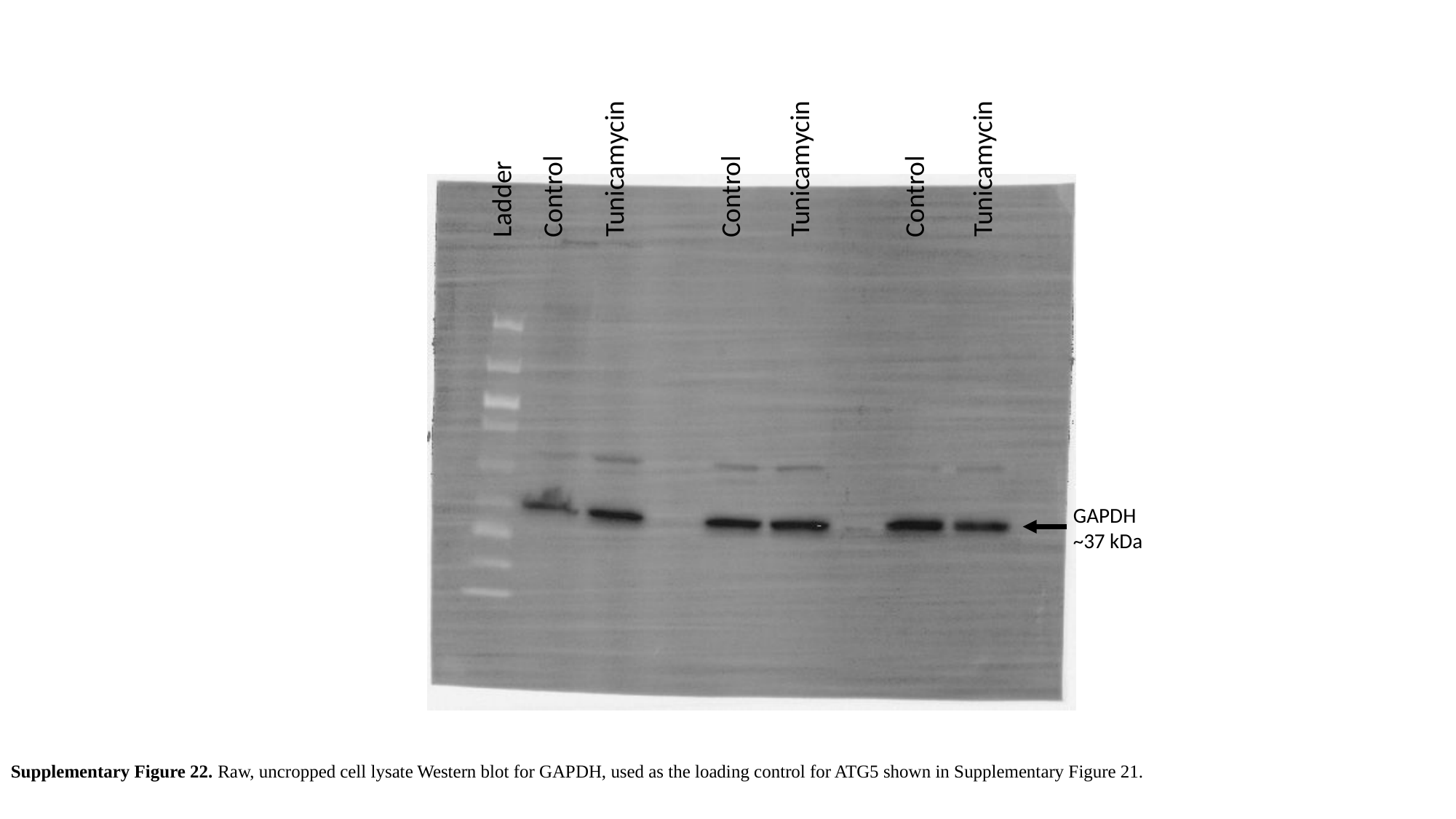

Tunicamycin
Tunicamycin
Tunicamycin
Control
Control
Control
Ladder
GAPDH
~37 kDa
Supplementary Figure 22. Raw, uncropped cell lysate Western blot for GAPDH, used as the loading control for ATG5 shown in Supplementary Figure 21.

## Slide 23
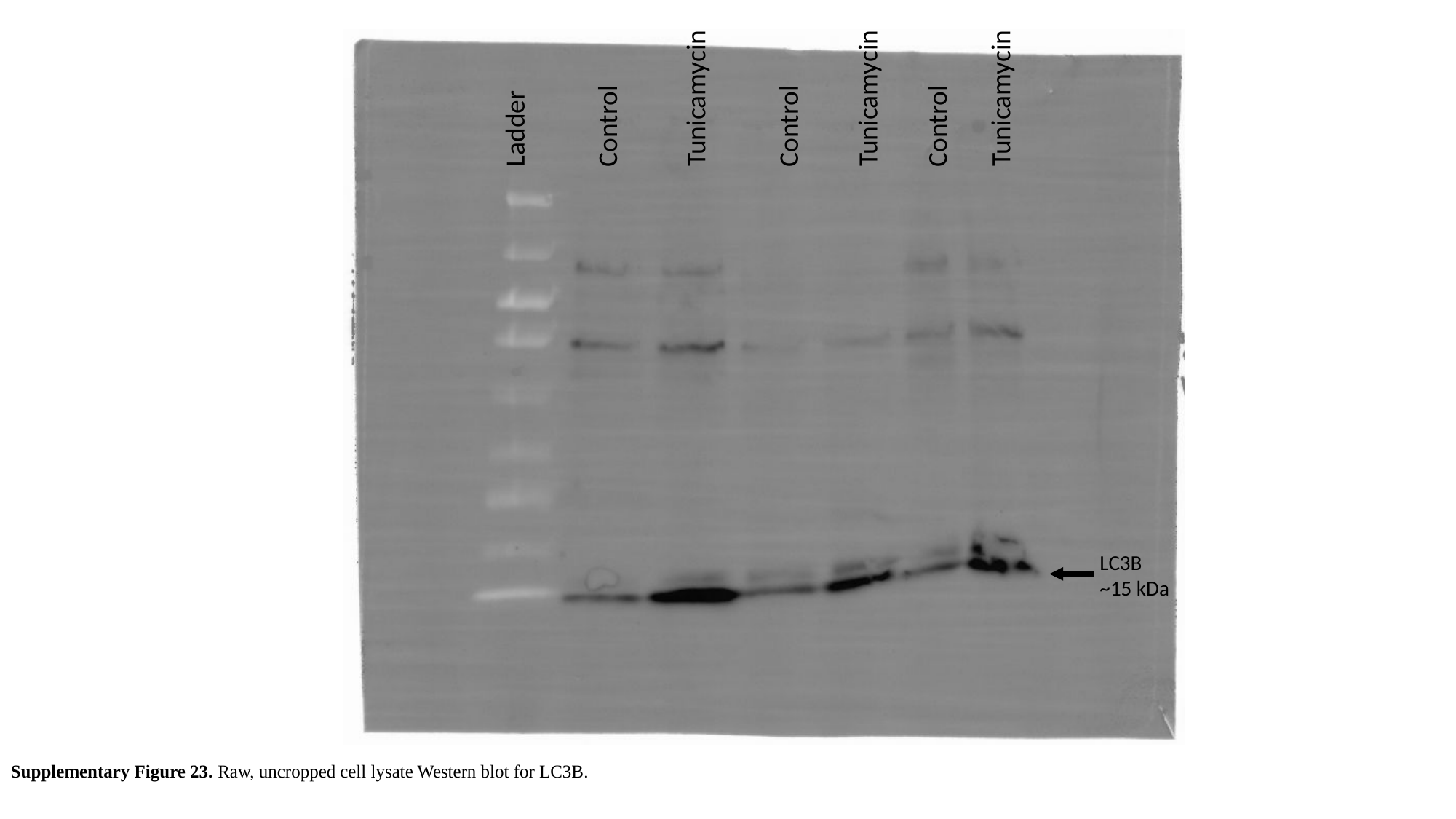

Tunicamycin
Tunicamycin
Tunicamycin
Control
Control
Control
Ladder
LC3B
~15 kDa
Supplementary Figure 23. Raw, uncropped cell lysate Western blot for LC3B.

## Slide 24
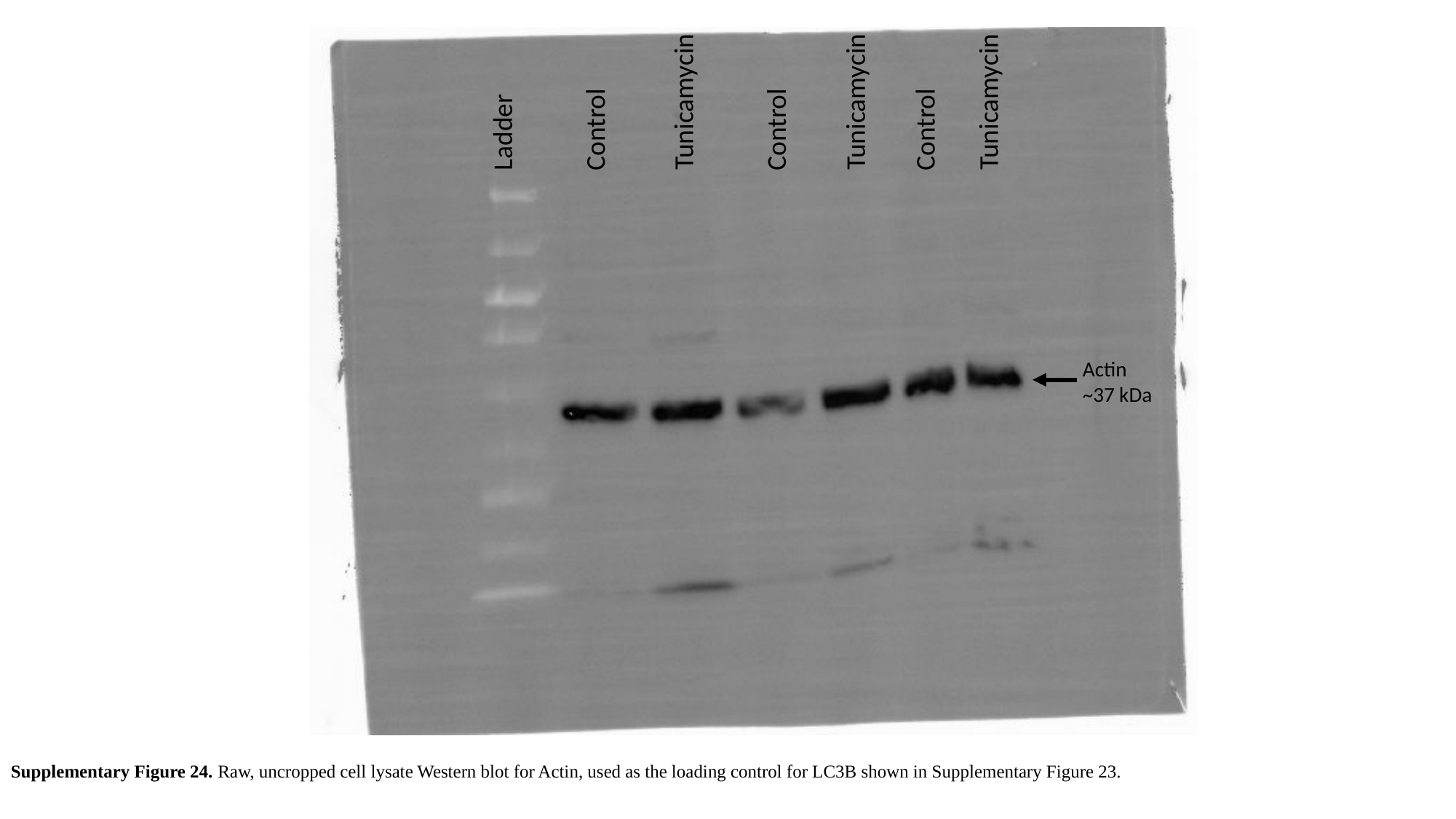

Tunicamycin
Tunicamycin
Tunicamycin
Control
Control
Control
Ladder
Actin
~37 kDa
Supplementary Figure 24. Raw, uncropped cell lysate Western blot for Actin, used as the loading control for LC3B shown in Supplementary Figure 23.

## Slide 25
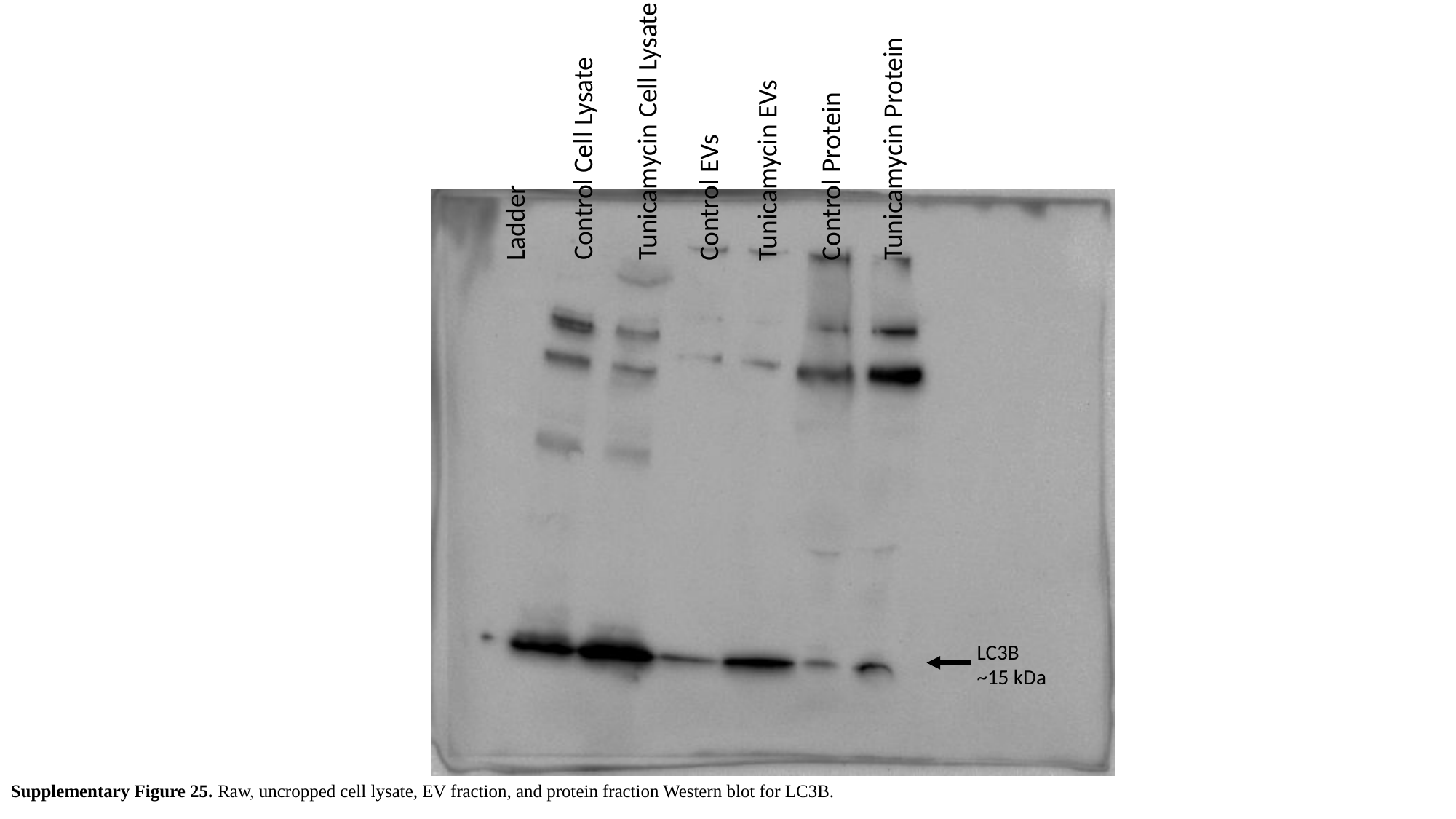

Tunicamycin Cell Lysate
Tunicamycin Protein
Control Cell Lysate
Tunicamycin EVs
Control Protein
Control EVs
Ladder
LC3B
~15 kDa
Supplementary Figure 25. Raw, uncropped cell lysate, EV fraction, and protein fraction Western blot for LC3B.

## Slide 26
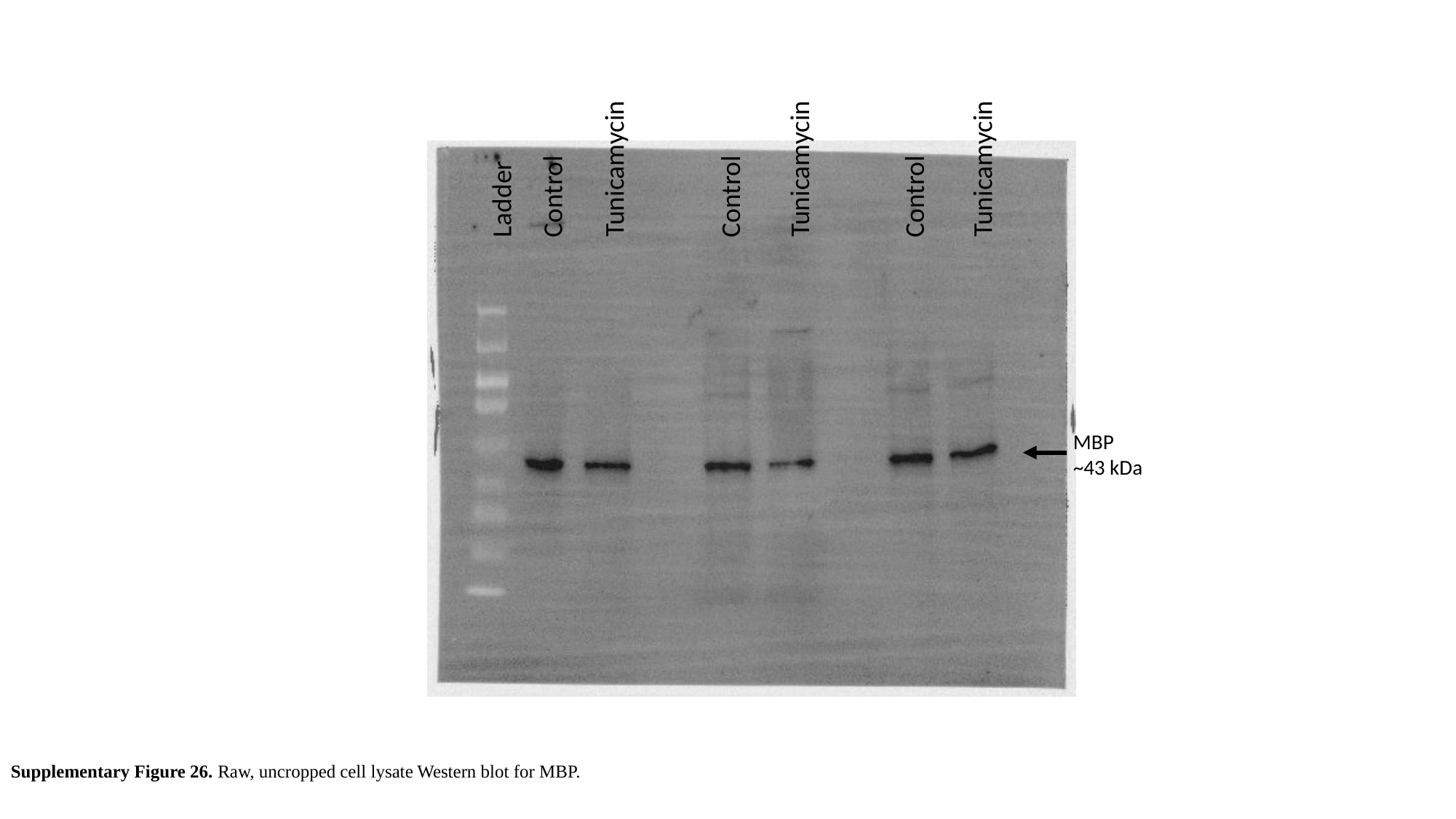

Tunicamycin
Tunicamycin
Tunicamycin
Control
Control
Control
Ladder
MBP
~43 kDa
Supplementary Figure 26. Raw, uncropped cell lysate Western blot for MBP.

## Slide 27
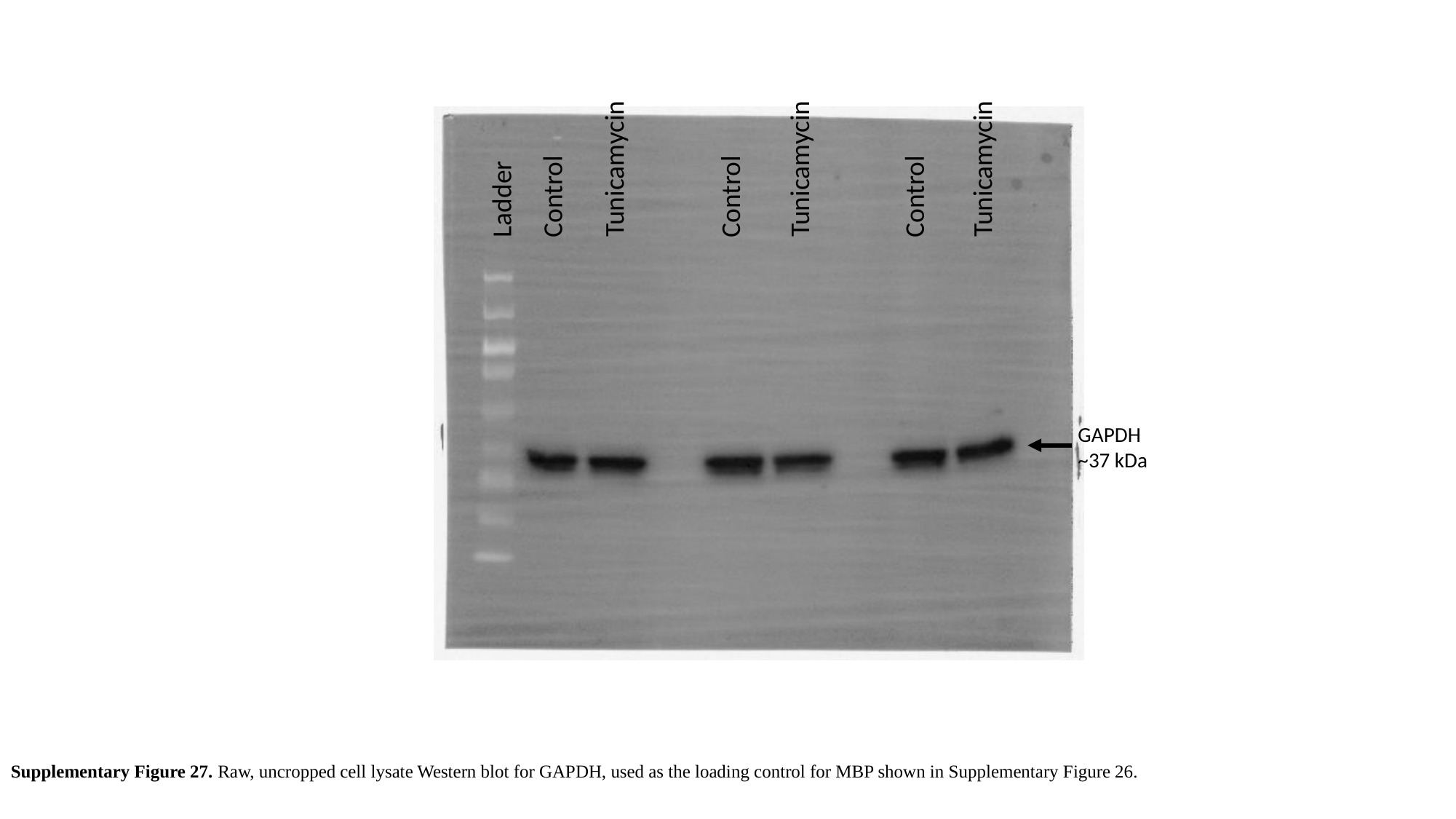

Tunicamycin
Tunicamycin
Tunicamycin
Control
Control
Control
Ladder
GAPDH
~37 kDa
Supplementary Figure 27. Raw, uncropped cell lysate Western blot for GAPDH, used as the loading control for MBP shown in Supplementary Figure 26.

## Slide 28
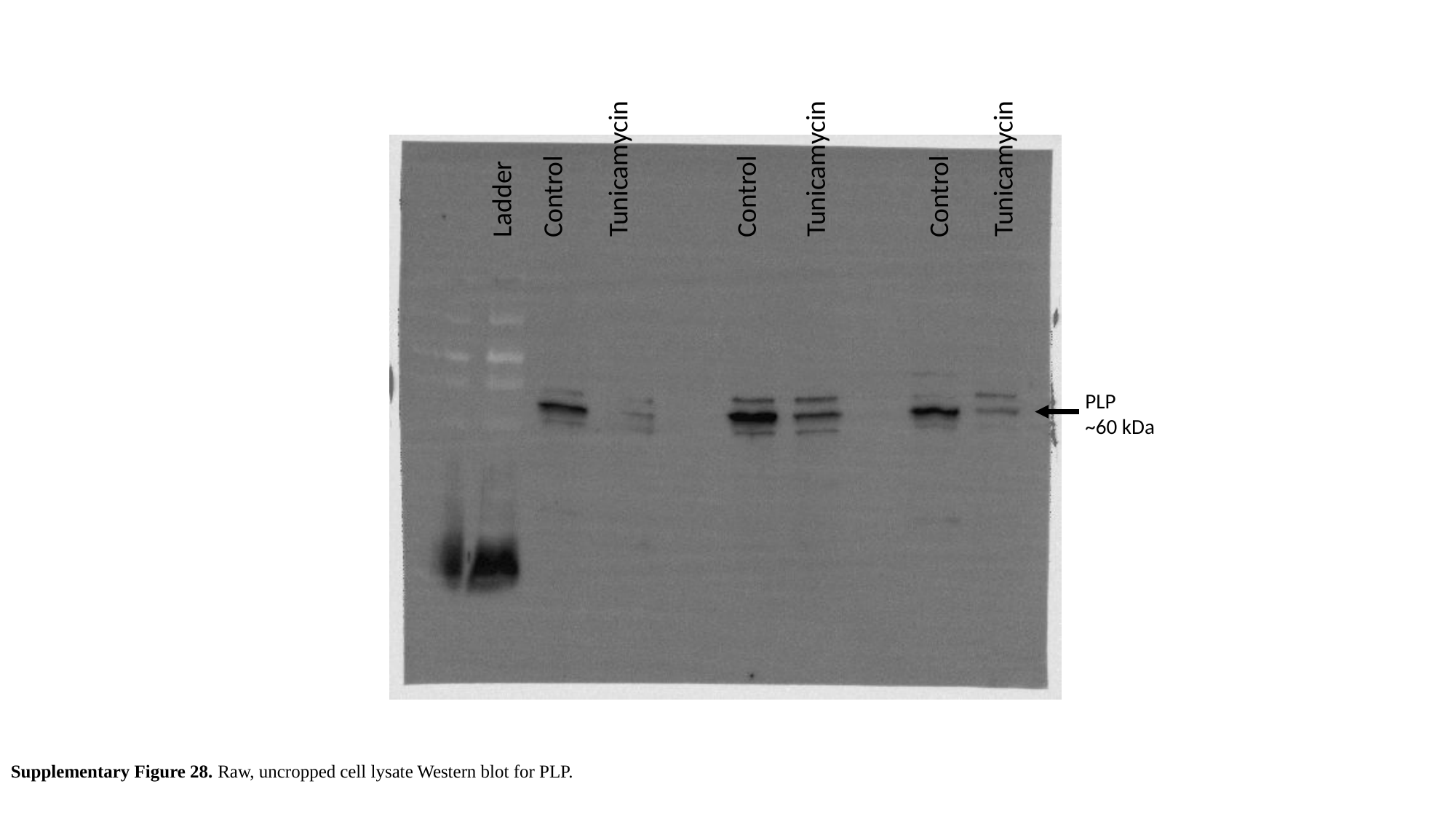

Tunicamycin
Tunicamycin
Tunicamycin
Control
Control
Control
Ladder
PLP
~60 kDa
Supplementary Figure 28. Raw, uncropped cell lysate Western blot for PLP.

## Slide 29
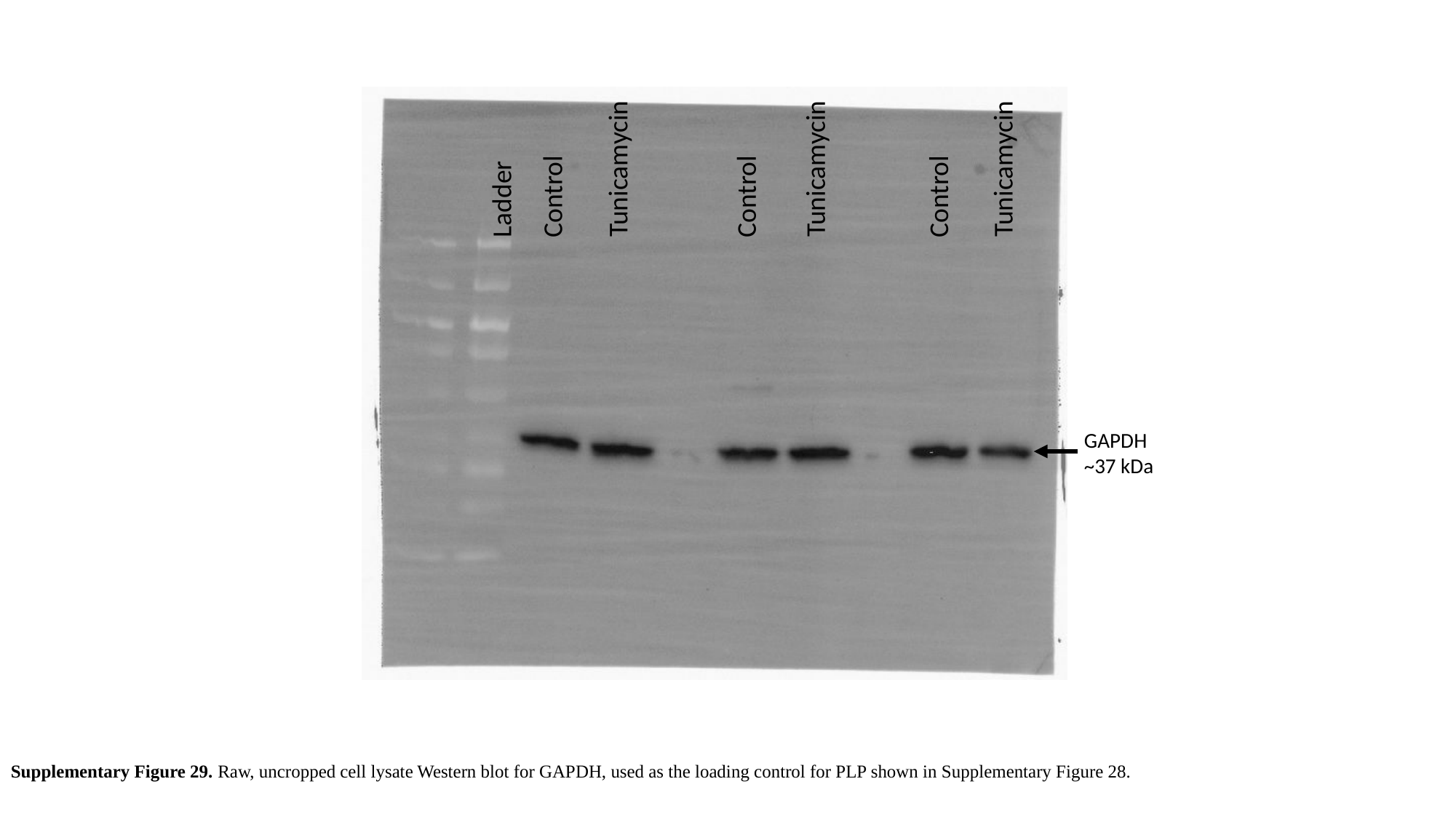

Tunicamycin
Tunicamycin
Tunicamycin
Control
Control
Control
Ladder
GAPDH
~37 kDa
Supplementary Figure 29. Raw, uncropped cell lysate Western blot for GAPDH, used as the loading control for PLP shown in Supplementary Figure 28.

## Slide 30
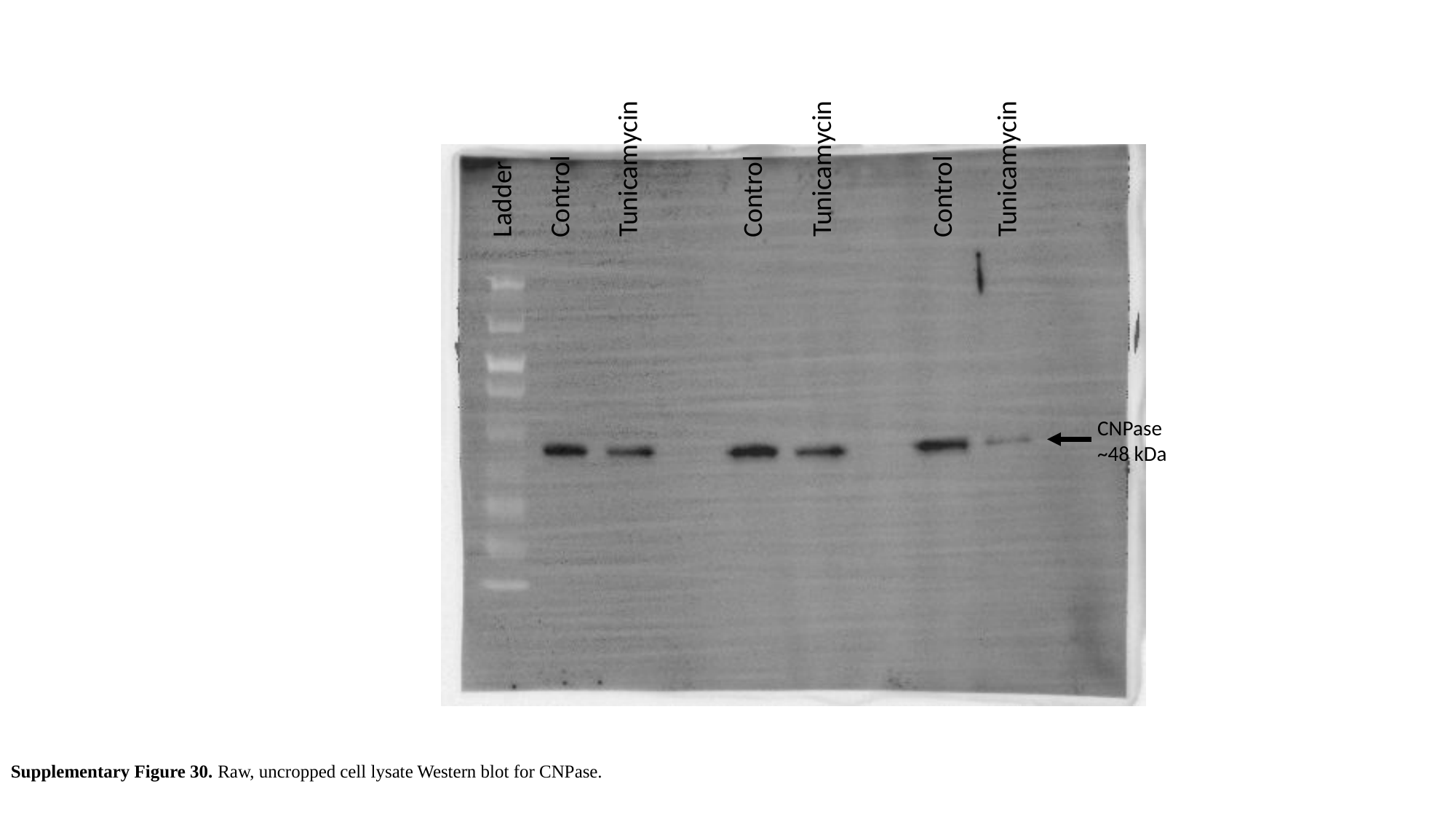

Tunicamycin
Tunicamycin
Tunicamycin
Control
Control
Control
Ladder
CNPase
~48 kDa
Supplementary Figure 30. Raw, uncropped cell lysate Western blot for CNPase.

## Slide 31
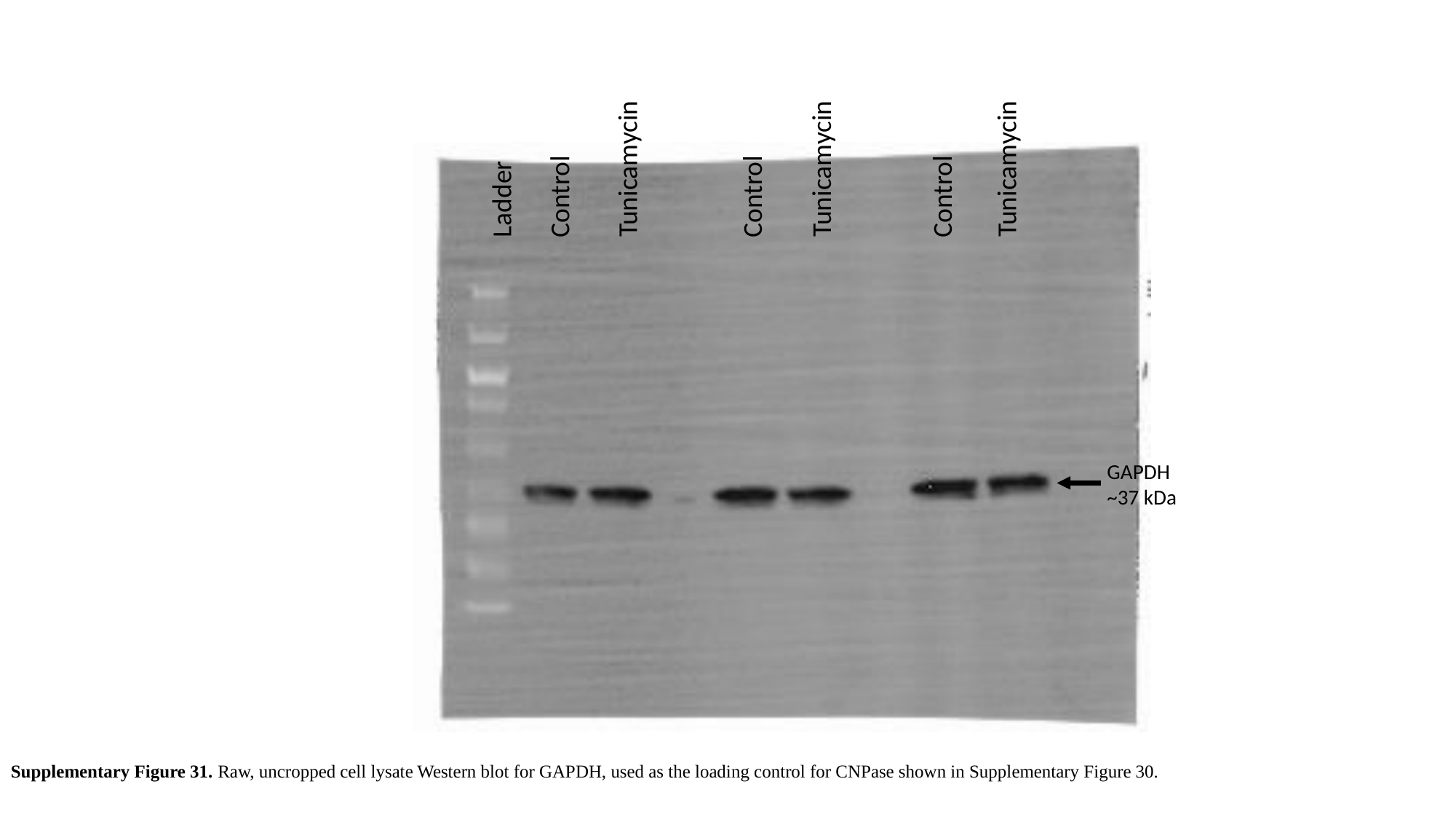

Tunicamycin
Tunicamycin
Tunicamycin
Control
Control
Control
Ladder
GAPDH
~37 kDa
Supplementary Figure 31. Raw, uncropped cell lysate Western blot for GAPDH, used as the loading control for CNPase shown in Supplementary Figure 30.
